# Purified Lotus Leaf Flavonoids in Lysozyme–Chitosan Coatings: Effects on Coating Properties and Strawberry Preservation

**DOI:** 10.3390/foods15173073

**Published:** 2026-08-30

**Authors:** Xinrui Gao, Lei Xiong, Dexin Zhang, Xi Feng, Huiqing Wu, Wen Huang, Zhili Ma

**Affiliations:** 1 School of Laboratory Medicine, Hubei University of Chinese Medicine, Wuhan 430065, China; gxr5682023@163.com (X.G.); 13687115630@163.com (L.X.); dexzhang@hbucm.edu.cn (D.Z.); huiqingwu2022@163.com (H.W.); 2 Hubei Shizhen Laboratory, Wuhan 430065, China; 3 Department of Nutrition, Food Science, and Packaging, San Jose State University, San Jose, CA 95192, USA; xi.feng@sjsu.edu; 4 College of Food Science and Technology, Huazhong Agricultural University, Wuhan 430070, China; huangwen@mail.hzau.edu.cn

**Keywords:** lotus leaf flavonoids, lysozyme–chitosan coating, edible coating, strawberry preservation, postharvest quality, antioxidant activity, antibacterial activity

## Abstract

Conventional lysozyme–chitosan (LCS) coatings combine the film-forming properties of chitosan with lysozyme-mediated antibacterial activity, but their antioxidant capacity and activity against Gram-negative bacteria remain limited. In this study, purified lotus leaf flavonoids (LLF) were incorporated into a LCS matrix at 0.1–0.3% (*w*/*v*) to examine how a defined flavonoid fraction affects matrix structure, film performance, and strawberry preservation under ambient storage. LLF showed strong radical-scavenging activity, with IC_50_ values of 6.244 µg/mL for DPPH and 2.146 µg/mL for ABTS, and inhibited *Staphylococcus aureus* and *Escherichia coli* (MIC values of 0.625 and 1.25 mg/mL, respectively). Structural analyses indicated concentration-dependent, predominantly non-covalent interactions between LLF and the LCS network. LLF addition increased color intensity and opacity and altered water-related, barrier, thermal, and mechanical properties, revealing a formulation trade-off rather than uniform improvement of all material attributes. The LLF-LCS systems nevertheless showed stronger antioxidant and in vitro antibacterial activities than the CS and LCS controls. During 6 d of storage at 25 ± 2 °C, 0.2–0.3% LLF-LCS treatments reduced decay, limited weight loss, retained firmness, and better-preserved ascorbic acid, total phenolics, and anthocyanins. These findings demonstrate a formulation-dependent structure–property–preservation relationship for purified LLF in LCS coatings under short-term ambient storage and identify 0.2% LLF as the most balanced formulation within the tested range.

## 1. Introduction

Petroleum-based food packaging has been extensively used because of its favorable properties for maintaining food quality over extended periods. However, its environmental persistence has raised growing concerns about microplastic pollution and non-renewable resource consumption [1,2]. These issues have stimulated continuing interest in biodegradable materials that can also actively contribute to food preservation.

Chitosan (CS) is one of the most widely studied biopolymers for food coatings because of its biodegradability, biocompatibility, safety, and film-forming ability [3,4]. CS-based coatings have been reported to delay postharvest deterioration of fruits and vegetables, although their effectiveness can be limited by moisture sensitivity and insufficient functional activity under practical storage conditions [5]. Lysozyme (LY), a natural antimicrobial enzyme capable of hydrolyzing β-1,4-glycosidic linkages in peptidoglycan, is particularly effective against Gram-positive bacteria [6]. For this reason, incorporating LY into CS to form lysozyme–chitosan composites (LCS) has become a common strategy to improve antimicrobial performance, and LCS-based films and coatings have been investigated in dairy products, seafood, vegetables, and fruits [7,8,9]. Nevertheless, conventional LCS systems still show important limitations for postharvest preservation, especially modest antioxidant capacity and incomplete antibacterial coverage, particularly against Gram-negative bacteria [10]. Therefore, introducing a suitable natural active component into the LCS matrix may be an effective way to broaden its functionality.

Plant polyphenols are frequently incorporated into CS-based films and coatings to enhance antioxidant protection and complement antimicrobial effects. For example, tea-polyphenol nanoparticles incorporated into CS films improved barrier, mechanical, antibacterial, and antioxidant properties and extended strawberry storage life [11]. More recently, a multicomponent coating containing LY, epigallocatechin gallate, and perilla extract was developed for strawberry preservation [12]. These studies demonstrate the potential of combining CS or LY with plant-derived compounds, but they also show that this is already an established strategy. In nanoparticle-based or multicomponent formulations, the preservation effect may arise from several active ingredients and processing steps, making it difficult to determine the contribution of an individual plant fraction.

Lotus (*Nelumbo nucifera*) is cultivated extensively in Asia, and lotus leaves are generated in large quantities but remain underutilized in many production regions [13,14]. Lotus leaf flavonoids (LLF), which mainly include quercetin, kaempferol, and their glycosides, have been reported to possess antioxidant and antibacterial activities [15]. Lotus leaf extract has already been incorporated into a polysaccharide composite coating for fresh goji fruit, where it reduced decay and weight loss and helped maintain postharvest quality [16]. However, that study used a crude extract in an alginate–konjac glucomannan-starch matrix, and therefore did not establish how a purified lotus leaf flavonoid fraction interacts with a LY-containing CS matrix. Our previous study examined purified LLF in zein/CS films and focused on film properties and solvent-responsive release [17], but it did not include LY or assess fruit preservation. Therefore, the behavior of purified LLF in an LCS coating and its relationship with postharvest fruit preservation have not yet been established.

Although LCS coatings and polyphenol-containing coatings have been widely studied, it remains unclear how a purified and chemically characterized lotus leaf flavonoid fraction affects the structure and performance of an LCS matrix and whether these changes translate into improved strawberry preservation. The major flavonoid constituents of LLF were first characterized by UPLC-QTOF-MS/MS, and their relative abundances were estimated from normalized peak areas. The characterized LLF fraction was subsequently incorporated into the LCS coating system at three concentrations, with CS and LCS included as separate controls. The resulting structural, optical, water-related, barrier, thermal, mechanical, antibacterial, and antioxidant properties were examined together with strawberry quality during ambient storage. We hypothesized that LLF would enhance the antioxidant activity and complement the antibacterial action of LCS, while its interaction with the matrix would produce concentration-dependent changes in film properties. Consequently, preservation performance was expected to depend on the balance between increased bioactivity and maintenance of suitable material properties.

## 2. Materials and Methods

### 2.1. Materials

CS (CAS: 9012-76-4; molecular weight ~200,000 Da; degree of deacetylation 95%; viscosity ≤ 200 cps), glycerol (≥99%), LY (egg white, ≥20,000 U/mg), DPPH, ABTS, oxalic acid, 2,6-dichlorophenolindophenol sodium salt (AR, 98%), and rutin standard (≥98%) were sourced from Shanghai Yuanye Biotechnology Co., Ltd. (Shanghai, China). LB Broth and LB Agar were obtained from Solarbio Science & Technology Co., Ltd. (Beijing, China). *Escherichia coli* ATCC 25922 and *Staphylococcus aureus* ATCC 6538 were supplied by the College of Food Science and Technology, Huazhong Agricultural University. Acetic acid and all other reagents were analytical grade. Fresh strawberries (*Fragaria ananassa* cv. Benihoppe), 25 ± 5 g each, were selected on the experimental day. LLF was obtained by solvothermal extraction and FL-3 macroporous resin purification, with the purified fraction containing 796.92 ± 16.76 mg rutin equivalents/g.

### 2.2. Characterization and Bioactivities of LLF

#### 2.2.1. UPLC-QTOF-MS/MS Characterization of LLF

LLF was characterized by UPLC-QTOF-MS/MS. Briefly, 20 mg of LLF was extracted with 200 μL of 75% methanol, ultrasonicated for 30 min, and centrifuged at 17,000× *g* for 10 min at 20 °C. The supernatant was subjected to LC-MS analysis. Separation was performed on a Waters H-Class UPLC system equipped with an ACQUITY UPLC HSS T3 column (1.8 μm, 2.1 × 100 mm) at 40 °C. The mobile phases were 0.1% formic acid in water (A) and 0.1% formic acid in acetonitrile (B), with a flow rate of 0.4 mL/min and an injection volume of 5 μL. The gradient was 95% A at 0–1.5 min, 90% A at 2.5 min, 60% A at 14 min, 5% A at 24–27 min, and 95% A at 27.1–30 min.

Mass spectra were acquired on an AB SCIEX 5600 Triple TOF system in both positive and negative electrospray ionization modes. The MS1 scan range was *m*/*z* 50–1200, and MS/MS spectra were acquired at a collision energy of 40 eV. Source settings were 55 psi for GS1 and GS2, 35 psi for curtain gas, 550 °C, and spray voltages of +5500 and −4500 V for positive and negative modes, respectively. Data were processed using MS-DIAL 4.6, and compounds were annotated by matching accurate mass and MS/MS spectra against METLIN, MassBank, MoNA, and HMDB. Mass tolerances were 0.01 Da for MS1 and 0.05 Da for MS2. Relative abundances were estimated from normalized peak areas within each ionization mode.

#### 2.2.2. Antioxidant Activity of LLF

The antioxidant activity of LLF was evaluated by DPPH and ABTS radical-scavenging assays with minor modifications based on the methods of Williams et al. [18] and Re et al. [19]. The stock solution of LLF was prepared at a concentration of 0.1 mg/mL. For the first test, the LLF was mixed in working solution, maintained in dark at room temperature for about 30 min, and the absorbance was recorded at 517 nm. For the second test, ABTS was reacted with potassium persulfate under dark conditions in order to generate ABTS radical cation. After 12 h incubation, it was diluted so that its absorbance would be 0.700 ± 0.02 at 734 nm. In the next step, LLF was mixed with this solution, allowed to react in dark at room temperature for 15 min, and absorbance was recorded at 734 nm. Scavenging activity was calculated by the equation:

(1) $$Scavening\;activity\left(\%\right)=1-\frac{A_{1}-A_{2}}{A_{0}}\times 100$$

where A_1_ is the absorbance of the reaction mixture containing both sample and radical solution, A_2_ is the absorbance of the sample blank, and A_0_ is the absorbance of the radical control.

#### 2.2.3. Antibacterial Activity of LLF

Antibacterial activity of LLF against *S. aureus* and *E. coli* was evaluated using agar diffusion and broth microdilution assays. Bacterial inocula were standardized to the required cell densities using an OD_600_-CFU calibration curve established prior to testing.

For the agar-diffusion assay, bacterial suspensions (10^8^ CFU/mL) were evenly spread on LB agar plates. Sterile Oxford cups were placed on the agar surface, and 200 μL of LLF solution at each tested concentration was added. Sterile water, which was used to prepare the LLF solutions, was included as the negative control. The plates were incubated at 37 °C for 24 h, after which the inhibition-zone diameters were measured in millimeters (*n* = 3).

Minimum inhibitory concentration (MIC) and minimum bactericidal concentration (MBC) were determined by using two-fold broth microdilution in 96-well plates according to CLSI guidelines. Serial dilutions of LLF in LB broth were made (40 to 0.3125 mg/mL) and inoculated with bacterial culture (10^6^ CFU/mL). The incubation was done at 37 °C for 24 h. MIC was defined as the lowest concentration of LLF showing no growth. MBC was determined by plating aliquots from wells with concentrations higher and lower than the MIC on LB agar. Incubation was done at 37 °C for 12 h.

### 2.3. Film Preparation

Film preparation was adapted from our previous method for LLF–biopolymer composites, with modifications tailored to the LCS matrix [17]. CS was dissolved in 2% (*w*/*v*) acetic acid, with glycerol (0.6%, *w*/*v*) added as a plasticizer, and the mixture was stirred for 30 min. LY was mixed with CS to prepare LCS composite (10% *w*/*w* LY to CS). LLF was added at 0.1%, 0.2%, and 0.3% (*w*/*v*) final concentrations to make 0.1% LLF-LCS, 0.2% LLF-LCS, and 0.3% LLF-LCS, respectively. The formulations were thoroughly mixed and sonicated, resulting in CS, LCS, 0.1% LLF-LCS, 0.2% LLF-LCS, and 0.3% LLF-LCS groups. Then, 70 mL of each composite was poured into 15 cm Petri dishes, dried at 40 °C for 12 h, conditioned at 25 °C and 53% relative humidity for about 48 h and kept at 4 °C until further use in fruit storage studies.

### 2.4. Characterization of Bioactive Films

#### 2.4.1. Microstructural and Structural Analyses

The surface morphology of the films was examined using scanning electron microscopy (SEM; Sigma 300, Carl Zeiss Microscopy GmbH, Jena, Germany). Film specimens measuring 10 mm × 10 mm were mounted on sample holders, sputter-coated with gold, and observed at an accelerating voltage of 3.0 kV.

The functional-group environment and possible matrix interactions in the films were evaluated by attenuated total reflectance Fourier transform infrared spectroscopy (ATR-FTIR; ALPHA II, Bruker Optik GmbH, Ettlingen, Germany). Spectra were recorded over the range of 4000–600 cm^−1^ at a resolution of 4 cm^−1^, with 32 scans co-added for each spectrum. Spectra were converted to absorbance when necessary and baseline-corrected using the asymmetric least-squares method with identical parameters for all samples (λ = 1 × 10^6^, *p* = 0.001, 20 iterations). The corrected spectra were displayed on a common intensity scale without vertical offset or individual normalization.

The O-H/N-H stretching region at 3500–3100 cm^−1^ was evaluated using the band maximum, full width at half maximum (FWHM), and integrated area. To reduce the influence of differences in overall spectral intensity and ATR contact, the integrated area of this region was expressed relative to that of the fingerprint region at 1800–900 cm^−1^. The C-H stretching region at 3000–2800 cm^−1^ was fitted using a linear local baseline and three Gaussian components assigned approximately to CH_2_ asymmetric stretching near 2920 cm^−1^, a possible CH_3_ stretching contribution near 2875 cm^−1^, and CH_2_ symmetric stretching near 2850 cm^−1^. The derived parameters were used only as descriptive, semi-quantitative spectral indices and were not interpreted as absolute measures of hydrogen-bond content or molecular CH_3_/CH_2_ ratios. In accordance with the instrumental resolution, band positions are reported as whole wavenumbers.

The crystalline characteristics of the films were examined by X-ray diffraction (XRD; D2, Bruker, Ettlingen, Germany) over a 2θ range of 5–50° at a scanning rate of 4° min^−1^.

#### 2.4.2. Thickness, Optical Properties and Surface Color

The measurements of thickness at eight arbitrary points were performed by the micrometer (Model 211-101K, Dongguan Sanliang Measuring Tools Co., Ltd., Dongguan, China), and the mean value was applied to calculate other parameters. The color coordinates L*, a*, and b* of the films were measured using a colorimeter (NR110, 3 nh Technology Co., Ltd., Shenzhen, China), with L_0_*, a_0_*, and b_0_* representing the corresponding coordinates of the reference standard. Further, the calculation of ΔE, WI, and YI was carried out using Equations (2)–(4). For opacity, the formula given in Equation (5) was applied where A600 represents absorbance and d is the thickness (mm).

(2) $$\Delta E=\sqrt{{\left(L^{*}-L_{0}^{*}\right)}^{2}+{\left(a^{*}-a_{0}^{*}\right)}^{2}+{\left(b^{*}-b_{0}^{*}\right)}^{2}}$$

(3) $$WI=100-\sqrt{{\left(100-L^{*}\right)}^{2}+{\left(a^{*}\right)}^{2}+{\left(b^{*}\right)}^{2}}$$

(4) $$YI=\frac{142.86\times b^{*}}{L^{*}}$$

(5) $$O=\frac{A600}{d}$$

#### 2.4.3. Moisture Content (MC), Water Solubility (WS) and Water Contact Angle (WCA)

MC and WS were determined according to a previously reported procedure with minor modifications [20]. Film specimens (2 cm × 2 cm) were weighed to obtain the initial mass (m_0_), dried at 105 °C for 24 h to constant weight (m_1_), immersed in 30 mL ultrapure water for 24 h, and then dried again at 105 °C for 24 h to obtain the final dry mass (m_2_). MC and WS were calculated using Equations (6) and (7), respectively. Surface wettability was evaluated by static water contact angle measurement using a contact angle goniometer (DSA30 S, KRÜSS GmbH, Hamburg, Germany). A 2 µL droplet of deionized water was deposited onto the film surface, and the contact angle was recorded.

(6) $$MC\left(\%\right)=\frac{m_{0}-m_{1}}{m_{0}}\times 100$$

(7) $$WS\left(\%\right)=\frac{m_{1}-m_{2}}{m_{1}}\times 100$$

#### 2.4.4. Water Vapor Permeability (WVP)

WVP was measured gravimetrically following the method of Zhang et al. [21]. Briefly, conical flasks containing 3 g anhydrous calcium chloride were sealed with the test films and placed in a desiccator maintained at 23 °C and 75% relative humidity for 48 h. The increase in flask mass (Δm) was attributed to water vapor transfer through the film. WVP was calculated using Equation (8):

(8) $$WVP=\frac{∆m\times d}{A\times t\times ∆P}$$

where Δm is the mass gain (kg), d is film thickness (m), A is the exposed permeation area (m^2^), t is the test time (s), and ΔP is the water vapor partial pressure difference across the film (2095 Pa).

#### 2.4.5. Oxygen Permeability (OP) and Carbon Dioxide Permeability (CDP)

Gas barrier properties were evaluated by the deoxidizer absorption method for oxygen and the alkali absorption method for carbon dioxide, as previously described [22]. Briefly, 3 g of deoxidizer and 5 g of potassium hydroxide were placed separately in 100 mL conical flasks, which were then sealed with the corresponding film samples. The assemblies were kept at 23 °C and 75% relative humidity for 48 h. OP and CDP were calculated according to the corresponding permeability equation, using film thickness, exposed area, exposure time, and the partial pressure difference in the target gas.

(9) $$P_{g}=\frac{\Delta m_{g}\times d}{A\times t\times \Delta P_{g}}$$

where *P_g_* represents OP or CDP for the corresponding gas, Δ*m_g_* is the mass change associated with absorption of the target gas, d is the film thickness, *A* is the exposed film area, *t* is the test time, and Δ*P_g_* is the partial-pressure difference in the corresponding gas across the film.

#### 2.4.6. Thermogravimetric Analysis (TGA)

Thermal stability of the films was evaluated using a thermogravimetric analyzer (STA200, Hitachi High-Tech Science Corporation, Tokyo, Japan). The analysis was conducted under a nitrogen atmosphere with a heating rate of 10 °C/min in the temperature range of 50–800 °C. Approximately 10 mg of each film sample was placed in an aluminum crucible, and an empty crucible was used as the reference. Thermogravimetric (TG) and derivative thermogravimetric (DTG) curves were obtained to assess weight loss behavior and thermal degradation characteristics.

#### 2.4.7. Mechanical Properties

The tensile strength (TS) and elongation at break (EB) of the films were measured using a texture analyzer (TA.XT Plus, Stable Micro Systems Ltd., Surrey, UK), following ASTM D882-18 [23]. Before analysis, film samples were equilibrated at 25 °C (53% relative humidity) for 48 h, cut into rectangular specimens of 60 mm × 10 mm with a gauge length of 30 mm, and then stretched at a constant speed of 0.5 mm/s until rupture. Six replicates were tested for each film formulation. TS and EB were calculated according to the following equation:

(10) $$TS\left(MPa\right)=\frac{F}{A}$$

where F is the maximum force at break (N) and A is the cross-sectional area of the film (mm^2^).

(11) $$B\left(\%\right)=\frac{L-L_{0}}{L_{0}}\times 100\%$$

where L is the elongation at break (mm) and L_0_ is the initial gauge length (mm).

#### 2.4.8. Determination of Antibacterial and Antioxidant Activities

The biological activity of the film-forming system was evaluated in terms of antibacterial and antioxidant properties.

Antibacterial activity was assessed by agar diffusion assay with minor modifications based on [24]. Suspensions of *Staphylococcus aureus* and *Escherichia coli* were adjusted to 10^8^ CFU/mL and evenly spread onto LB agar plates using sterile cotton swabs. Wells with a diameter of 9 mm were then punched in the agar, and the corresponding film-forming solutions were added. After incubation at 37 °C for 24 h, inhibition zone diameters were measured in millimeters.

For antioxidant analysis, film samples were extracted with methanol for 12 h, and the supernatants were collected for DPPH and ABTS radical-scavenging assays according to [21] with minor modifications. In the DPPH assay, 20 μL of film extract was mixed with 180 μL of 0.1 mM DPPH solution and incubated in the dark for 30 min before the absorbance was recorded at 517 nm. In the ABTS assay, the radical solution was prepared by reacting ABTS stock solution with potassium persulfate in the dark for 12 h and then diluting it to an absorbance of 0.70 ± 0.02 at 734 nm. A 20 μL aliquot of film extract was then mixed with 180 μL of the ABTS working solution, kept in the dark for 15 min, and measured at 734 nm.

(12) $$DPPH\;scavenging\;activity\left(\%\right)=1-\frac{A_{1}-A_{2}}{A_{0}}\times 100$$

(13) $$ABTS\;scavenging\;activity\left(\%\right)=1-\frac{A_{1}-A_{2}}{A_{0}}\times 100$$

where A_1_ is the absorbance of the test sample, A_2_ is the absorbance of the sample blank, and A_0_ is the absorbance of the control.

### 2.5. Application in Strawberry Preservation

Strawberries harvested on the same day from a single harvest lot were selected for similar maturity, size, color, and initial mass. Fruits with visible mechanical damage or decay were excluded. The selected fruits were surface-sanitized by immersion in 2% (*v*/*v*) sodium hypochlorite solution for 30 s, thoroughly rinsed with running water, and air-dried for 2 h under ambient conditions.

A total of 192 strawberries were randomly assigned to six treatments: control, CS, LCS, 0.1% LLF-LCS, 0.2% LLF-LCS, and 0.3% LLF-LCS. Each treatment contained 32 fruits, with eight fruits allocated in advance to each sampling time on days 0, 2, 4, and 6. Control fruits were immersed in deionized water for 30 s, whereas fruits in the coating groups were immersed in the corresponding coating solutions for the same duration. After air-drying, all fruits were stored at 25 ± 2 °C and 55 ± 5% relative humidity. The complete storage trial was conducted once using fruit from the same harvest lot.

At each sampling time, weight loss and decay incidence were first evaluated for the eight fruits assigned to each treatment. Fruit mass was recorded immediately after coating and drying (m_0_) and again on the corresponding sampling day (m_i_; i = 2, 4, or 6), and weight loss was calculated as (m_0_ − m_i_)/m_0_ × 100. Decay incidence was assessed based on visible mycelial growth and/or soft rot and expressed as the percentage of decayed fruits among the eight fruits examined. Three of the eight fruits were then randomly selected for photography. Firmness was measured in the remaining five fruits at the fruit equator using a texture analyzer (TA.XT Plus, Stable Micro Systems, Godalming, UK) equipped with a 2 mm cylindrical probe, with a test speed of 2 mm·s^−1^ and a penetration depth of 2 mm. Three puncture measurements were obtained from each fruit and averaged to provide one firmness value per fruit.

Following these measurements, the coating layer was gently removed and all eight fruits from each treatment were cut into small pieces and combined. The pooled fruit tissue was divided by weight into three equal portions, which were homogenized separately for physicochemical analyses. Total soluble solids (TSS) were measured using a digital refractometer (PAL-1, ATAGO Co., Ltd., Tokyo, Japan), and pH was measured using a pH meter (pHS-3 C, LeiCi/INESA Scientific Instrument Co., Shanghai, China). Titratable acidity (TA) was determined by titration with 0.1 M NaOH and expressed as citric acid equivalents. Total phenolic content (TPC), total anthocyanin content (TAC), and ascorbic acid content (AAC) were determined using the Folin–Ciocalteu method, the pH differential method, and DCPIP titration according to GB 5009.86-2016, respectively, following the referenced procedures [25].

### 2.6. Statistical Analysis

All statistical analyses were performed using IBM SPSS Statistics 27.0. Data are presented as the mean ± standard deviation (SD). The sample size and experimental unit for each assay are specified in the corresponding methods, tables, and figure captions. Repeated measurements obtained from the same experimental unit were not treated as independent observations.

For the strawberry preservation trial, weight loss was determined using eight individual fruits per treatment at each sampling time (*n* = 8), and firmness was determined using five individual fruits (*n* = 5), with three puncture measurements averaged to obtain one value for each fruit. For TSS, pH, TA, TPC, TAC, and AAC, tissue from the eight fruits in each treatment was pooled and divided into three equal portions that were homogenized and analyzed separately. Treatment effects for fruit-level measurements were analyzed separately at each storage time by one-way ANOVA followed by Tukey’s post hoc test, with *p* < 0.05 considered statistically significant.

## 3. Results and Discussion

### 3.1. Chemical Characterization and Intrinsic Bioactivities of Purified LLF

UPLC-QTOF-MS/MS profiling revealed that the purified LLF fraction contained a series of flavonol glycosides and related flavonoids (Table 1 and Figure S1). In the negative-ion mode, quercetin 3-O-glucuronide (miquelianin) and peltatoside showed the highest relative peak areas, accounting for 17.89% and 12.35%, respectively, followed by quercetin 3-O-arabinoside (3.86%) and Luteolin (2.51%). In the positive-ion mode, prominent constituents included quercetin 3-O-glucuronide (miquelianin; 14.65%), kaempferol 3-O-glucoside (astragalin; 6.29%), quercetin 3-O-galactoside (hyperoside; 3.78%), and kaempferol (3.32%). Other detected flavonoids included isorhamnetin 3-O-galactoside, myricetin 3-O-galactoside, rutin, and isorhamnetin 3-O-rutinoside. Detailed mass-spectrometric annotations are provided in Table S1. Because peak-area normalization was performed separately for each ionization mode, these values represent mode-specific relative abundances rather than absolute concentrations.

**Figure 1:**
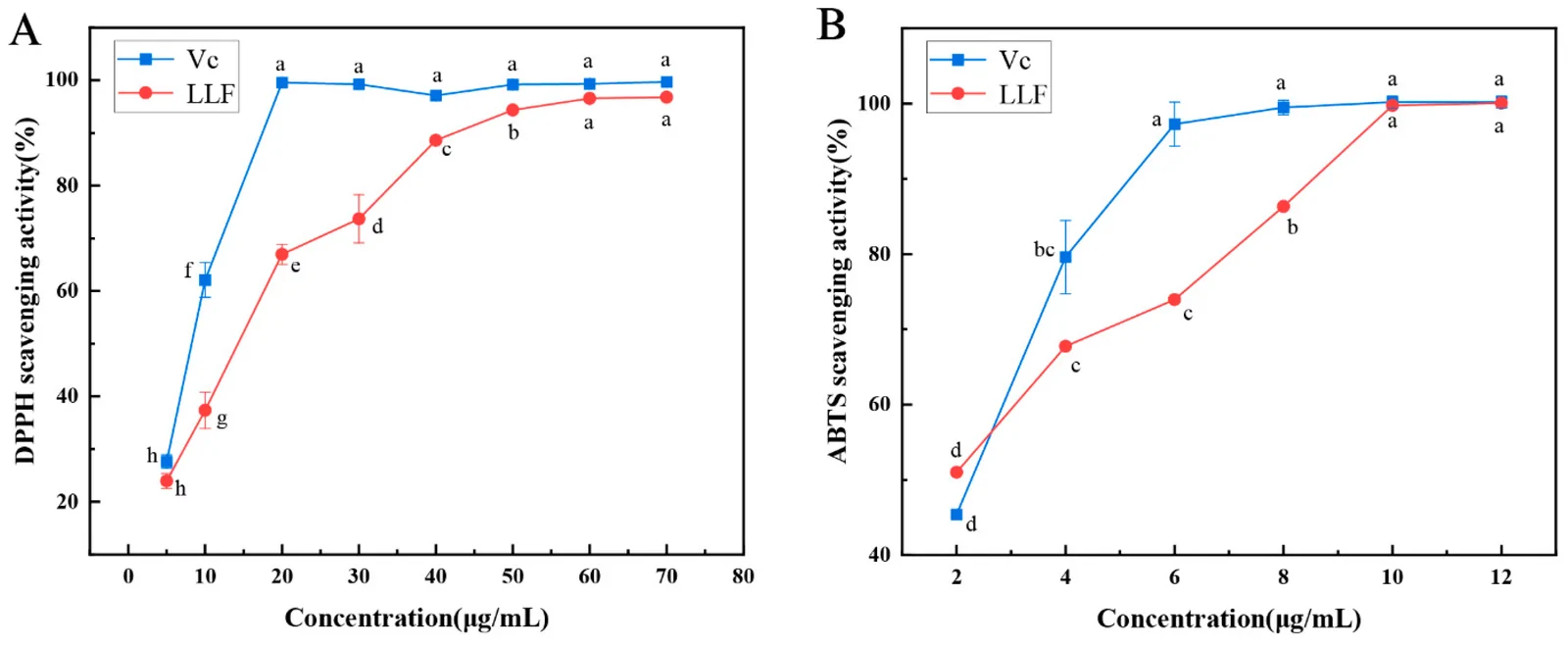
Radical-scavenging activity of LLF: (**A**) DPPH scavenging activity; (**B**) ABTS scavenging activity at different LLF concentrations. Vc was used as a positive control. Data are mean ± SD (*n* = 3). Different letters indicate significant differences (*p* < 0.05).

As shown in Figure 1, LLF exhibited pronounced radical-scavenging activity in both DPPH and ABTS assays, increasing in a dose-dependent manner over the tested concentration range. The IC_50_ values were 6.244 μg/mL for DPPH and 2.146 μg/mL for ABTS, whereas the corresponding values for ascorbic acid were 3.824 and 2.174 μg/mL, respectively. LLF therefore showed lower activity than ascorbic acid in the DPPH assay but a comparable response in the ABTS assay. The difference between the two assays may be attributed to their distinct radical characteristics, reaction media, and reaction kinetics, as well as their different sensitivities to phenolic structures. The relatively strong ABTS-scavenging response of LLF may be related to the presence of hydroxylated quercetin and kaempferol glycosides [26,27]. These results indicate that LLF has substantial radical-scavenging activity, although its apparent activity depends on the assay employed.

The OD_600_ CFU calibration curves showed good linearity for *S. aureus* (y = 0.0446 x + 0.0206, R^2^ = 0.9985) and *E. coli* (y = 0.1343 x + 0.0179, R^2^ = 0.9991). LLF produced concentration-dependent inhibition zones against both strains, with consistently larger zones against *S. aureus* (Table 2). Consistent with this pattern, *S. aureus* showed lower MIC and MBC values (0.625 and 5 mg/mL, respectively) than *E. coli* (1.25 and 20 mg/mL, respectively). The lower susceptibility of *E. coli* is consistent with previous findings that the outer membrane of Gram-negative bacteria may restrict the access of flavonoids and other phenolic compounds to cellular targets [28]. These results indicate that LLF inhibited both bacterial strains under the conditions tested, with greater activity against *S. aureus.* Together with its radical-scavenging activity, this antibacterial response supported the further evaluation of LLF as an active component in the LCS coating system.

### 3.2. Structural Characterization of the Films

#### 3.2.1. Surface Morphology and Microstructural Organization

**Figure 2:**
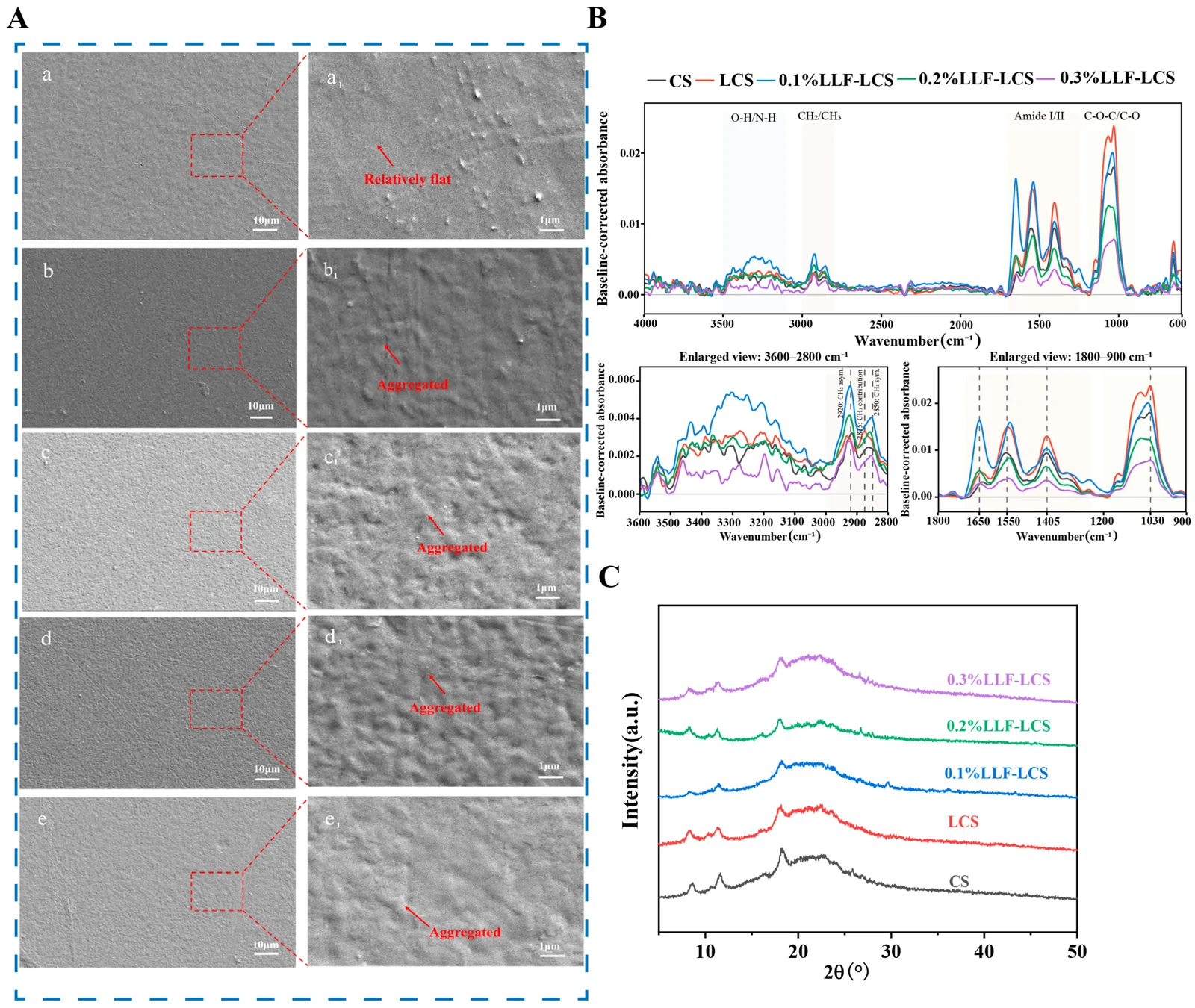
Microscopic characterization of films: (**A**) SEM images of the film surfaces at two magnifications: (**a**,**a_1_**) CS; (**b**,**b_1_**) LCS; (**c**,**c_1_**) 0.1% LLF-LCS; (**d**,**d_1_**) 0.2% LLF-LCS; and (**e**,**e_1_**) 0.3% LLF-LCS; (**B**) ATR-FTIR spectra after asymmetric least-squares baseline correction, presented in an overlapping layout without vertical offset or individual normalization. Shaded areas indicate the principal spectral regions, and enlarged views of the 3600–2800 and 1800–900 cm^−1^ ranges are included to improve visualization of overlapping features; (**C**) XRD patterns of the films.

The microscopic images in Figure 2A show different surface shapes for the CS films. CS film was relatively smooth and continuous, with occasional minor particles. After LY addition, LCS film exhibited scattered surface protuberances and heterogeneities, possibly due to local aggregation and microstructure change at composite formation. After LLF inclusion, surface shape changed based on concentration: at 0.2% LLF, the number of protrusions and heterogeneities increased in concentration, suggesting less homogeneous dispersions and microdomains. At 0.3% LLF, the film surface appeared comparatively compact, suggesting that LLF concentration affected its distribution and the surface organization of the LCS matrix. Similar patterns have been observed for CS–protein composite systems with plant polyphenols, where better intermolecular interactions lead to better phase compatibility and a more continuous film network [29,30].

#### 3.2.2. ATR-FTIR Characterization of Functional-Group Environments in the Composite Matrix

ATR-FTIR analysis was performed to examine changes in the functional-group environment of the films after the incorporation of LY and LLF (Figure 2B). The spectra were baseline-corrected using identical asymmetric least-squares parameters and are presented on a common scale without vertical offset or individual normalization. The CS film exhibited a broad band in the 3500–3100 cm^−1^ region, mainly arising from overlapping O-H and N-H stretching vibrations. Two bands at approximately 2920 and 2850 cm^−1^ were assigned to the asymmetric and symmetric C-H stretching vibrations of CH_2_ groups, respectively. Additional characteristic bands were observed near 1650, 1550, and 1405 cm^−1^ and within the 1150–1020 cm^−1^ region, corresponding to amide-related vibrations, C-H bending, and C-O-C/C-O stretching of the CS backbone [31,32].

After LY incorporation, the bands near 2920 and 2850 cm^−1^ remained visible, whereas the spectral profile between them changed. Three-component fitting of the 3000–2800 cm^−1^ region showed that the A_2875_/(A_2920_ + A_2850_) area index increased from 0.156 in CS to 0.230 in LCS (Table S2 and Figure S2). This result is consistent with an increased relative contribution from methyl-containing groups after LY incorporation. Accordingly, the change near 2875 cm^−1^ was interpreted as a modification of the relative CH_3_/CH_2_ spectral contribution rather than as a shift in the CH_2_ stretching band. Because of the substantial overlap among these bands and the model dependence of peak fitting, the calculated ratio was used only as a descriptive, semi-quantitative spectral index. Changes in the amide I and amide II regions near 1650 and 1550 cm^−1^ were also consistent with incorporation of LY and modification of the local environment of protein and polysaccharide functional groups [33].

The O-H/N-H stretching region was further evaluated using the band maximum, FWHM, and a relative integrated-area index (Table S2). The band maximum changed from 3361 cm^−1^ in CS to 3278 cm^−1^ in LCS, while the FWHM increased slightly from 310 to 318 cm^−1^. These differences are consistent with modification of the hydrogen-bonding environment after LY incorporation. Following LLF addition, the band position, FWHM, and relative area varied among the formulations without a monotonic relationship with LLF concentration. The O-H/N-H-to-fingerprint area index ranged from 0.095 to 0.143 among the composite films. These results are more consistent with formulation-dependent reorganization of the polar-group environment than with a progressive strengthening of hydrogen bonding.

LLF incorporation also modified the band shapes and relative intensities in the amide and polysaccharide fingerprint regions, particularly near 1650 cm^−1^ and within approximately 1060–1030 cm^−1^. Similar changes in the O-H/N-H and polysaccharide fingerprint regions have been reported after the incorporation of polyphenol-rich components into CS films [34,35]. Lotus leaf flavonoids are predominantly flavonol glycosides derived from quercetin and kaempferol, which contain multiple hydroxyl, carbonyl, and glycosidic C-O groups that may contribute to the overlapping FTIR bands in these regions [26,36]. These spectral responses varied among the formulations rather than increasing progressively with LLF concentration. The absence of new absorption bands suggests that LLF was incorporated into the LCS matrix without the formation of new covalent bonds. Instead, the observed changes in band position, shape, and relative intensity are consistent with a reorganization of the non-covalent interaction environment within the composite matrix, possibly involving hydrogen bonding.

#### 3.2.3. Crystalline Organization of the Composite Films

As shown in Figure 2C, CS displayed characteristic reflections at 2θ ≈ 8.3°, 11.3°, 18.1° and a broad halo centered at 21.7°, consistent with the semi-crystalline nature of CS and the coexistence of ordered domains and amorphous regions [37,38]. After LY incorporation, the LCS films exhibited changes in the diffraction profile, including the appearance of weak features near 10.5° and 21.0°, suggesting a rearrangement of CS crystalline domains and reduced lattice regularity upon composite formation [39]. Upon LLF addition, these features became less distinguishable, and an additional weak signal near ~30° was observed only at 0.1% LLF; this signal disappeared at higher LLF loadings. Overall, the progressive attenuation/broadening of characteristic reflections with increasing LLF content indicate that LLF incorporation tends to disrupt the ordered packing of CS chains and to promote a more amorphous composite structure, which is consistent with the matrix reorganization indicated by the FTIR results [40,41]. Notably, no additional crystalline phases were evident at higher LLF levels, suggesting that LLF did not form a distinct crystalline phase detectable by XRD at the higher concentrations.

### 3.3. Physicochemical Properties of the Films

#### 3.3.1. Optical Properties and Color Characteristics

As shown in Table 3, LY incorporation did not significantly affect film thickness or opacity, although the decrease in L* and increase in b* indicated a visible yellowing of the LCS film. The LLF-LCS films were significantly thicker than the CS film and showed marked changes in optical appearance. LLF incorporation decreased L* and increased a*, b*, ΔE, and YI relative to LCS, although the changes in individual color parameters were not strictly proportional to LLF concentration. In contrast, opacity increased progressively from 1.14 A·mm^−1^ for LCS to 1.38, 1.73, and 1.86 A·mm^−1^ for the 0.1%, 0.2%, and 0.3% LLF-LCS films, respectively. Comparable increases in film coloration and opacity have been reported after the incorporation of polyphenol-rich fractions into CS-based matrices [33,42]. These changes are generally associated with visible-light absorption by phenolic chromophores, together with increases in solid content and changes in the distribution of the active fraction within the polymer matrix. The higher opacity may reduce the transmission of visible light through the film; however, because UV-visible transmittance was not measured, the present results should not be interpreted as direct evidence of improved UV-barrier performance.

The pronounced color changes also have practical implications for the use of LLF-LCS as a fruit coating. The natural red color, brightness, and surface gloss of strawberries contribute to their perceived freshness and overall visual quality, and previous coating studies have included these attributes in sensory acceptance assessments [43,44]. Although the coloration imparted by LLF may be less pronounced when applied as a thin coating on strawberries than in freestanding films, excessive opacity could still modify the visual appearance of the fruit. Further sensory and instrumental color analyses are therefore needed to determine whether these changes influence consumer acceptance.

The increased color and opacity of the LLF-LCS films may alter the natural appearance and consumer acceptance of coated strawberries, as visual and sensory quality are important considerations in the evaluation of edible fruit coatings [43]. Because taste and odour were not assessed in the present study, their possible changes remain unknown. Moreover, the egg-white-derived LY may present an allergen concern for susceptible consumers, and applicable allergen-labelling requirements should be considered. Sensory evaluation and market-specific regulatory assessment of both LY and LLF are therefore required before commercial application.

#### 3.3.2. Barrier Properties Water-Vapor and Gas-Barrier Performance

Previous studies have shown that LY incorporation can modify the organization and mechanical behavior of CS films without necessarily changing their water-vapor barrier properties. Park et al. reported no significant change in WVP after LY addition, although the mechanical properties varied with LY content [45]. Consistent with this finding, the WVP of LCS (2.69 ± 0.12 × 10^−13^ kg·m·m^−2^·s^−1^·Pa^−1^) did not differ significantly from that of CS (2.78 ± 0.18 × 10^−13^ kg·m·m^−2^·s^−1^·Pa^−1^).

LLF incorporation produced an overall increase in WVP, although a significant difference was observed only at 0.3% LLF. Previous studies have reported both increases and decreases in WVP after the incorporation of plant-derived phenolic components into CS films [33,46,47], indicating that the barrier response depends on the structure, concentration, and mode of incorporation of the active compound. In the present system, the hydrophilic glycoside groups of LLF, together with changes in matrix organization indicated by the SEM and FTIR results, may have facilitated water transport at the highest loading.

OP remained statistically unchanged among the formulations, whereas CDP increased after LLF incorporation. Consequently, the CDP/OP ratio increased from 1.24 × 10^3^ for CS to 1.42 × 10^3^ for the 0.3% LLF-LCS film, indicating a modest change in relative gas transport. However, a modified-atmosphere effect cannot be inferred without direct measurements of headspace O_2_, CO_2_, and fruit respiration.

#### 3.3.3. Water Related Properties

Water-related properties (MC, WS and WCA) can be used to evaluate the water-sensitive properties of films to provide a basis for practical application of the films. As shown in Table 4, MC decreased from 29.63% in CS to 27.34% in LCS and further to 19.95–23.34% in the LLF-LCS films. This trend indicates lower overall moisture retention after conditioning and may be associated with changes in the availability of hydrophilic sites and the organization of the polymer–protein–flavonoid matrix. Interactions among CS, LY, and LLF may reduce the availability of some polar sites for water association, although the distribution of different water populations was not directly determined. However, the oven-drying method used for MC determination does not distinguish free water from weakly or strongly bound water.

In addition, the WS values of the LLF-LCS films were higher compared to the CS and LCS films, which can be attributed to the hydrophilicity of the LLF component. According to [36], LLF primarily consisted of quercetin glycosides, such as quercetin-3-O-galactoside, quercetin-3-O-glucoside, and kaempferol-3-O-glucoside. These compounds possessed multiple polar groups, including phenolic hydroxyl and glycosidic chains, which can form hydrogen bonds with water, enhancing their water solubility and hydrophilicity. The incorporation of LLF, therefore, improves the film’s ability to interact with water, thereby increasing the overall hydrophilicity and water solubility of the composite film. Importantly, the enhanced WS of the films commonly significantly improved their biodegradability [48,49].

According to Table 4, compared to the WCA values of CS films (90.67° ± 0.5°), the WCA values of LCS films significantly decreased to 73.27° ± 0.42° (*p* < 0.05). Brychcy et al. [50] reported that LY was uniformly distributed within the CS matrix, forming microscale “hydrophilic channels” or hydrophilic surfaces, which facilitate rapid interaction between the membrane and water. The WCA values of the LLF-LCS film series significantly increased with the addition of LLF (*p* < 0.05), with the highest WCA values observed in the 0.2% LLF-LCS films (88.43° ± 0.31°). The incorporation of the appropriate concentration of LLF enables it to interact with the hydrophilic groups within the film matrix, leading to a competitive effect that diminishes its interaction with water molecules. At the concentration of 0.3% LLF, saturation occurred in the binding of LLF to the hydrophilic groups in the film matrix, resulting in a decline in WCA values due to the inherent hydrophilic properties of LLF.

Overall, LLF incorporation modified the water sensitivity and surface wettability of the LCS coating matrix, while an appropriate LLF level improved surface hydrophobicity.

#### 3.3.4. Thermal Degradation Behavior and Low-Temperature Mass-Loss Characteristics

**Figure 3:**
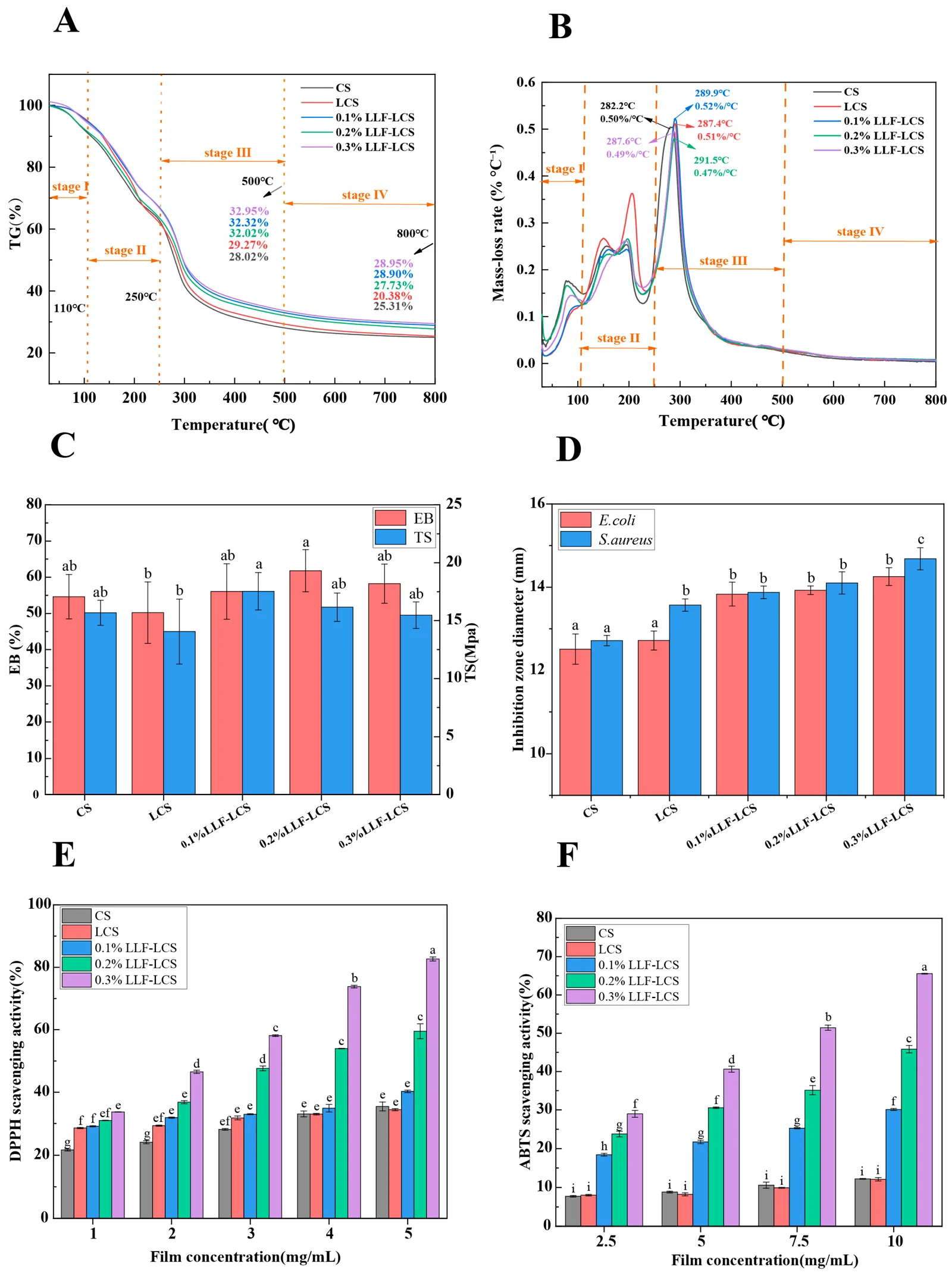
Thermal, mechanical, antibacterial, and antioxidant properties of the films. (**A**) TG curves and (**B**) DTG curves of the five film formulations. (**C**) Tensile strength (TS) and elongation at break (EB) (*n* = 6 film specimens). (**D**) Inhibition-zone diameters against *S. aureus* and *E. coli* (*n* = 3). (**E**) DPPH and (**F**) ABTS radical-scavenging activities of the film extracts (*n* = 3). Data in panels C-F are presented as mean ± SD. Different letters indicate significant differences among formulations under the same test condition (*p* < 0.05).

The thermal degradation behavior of the films was evaluated by TGA and DTG (Figure 3A,B). All samples exhibited a multistep mass-loss profile. To characterize their low-temperature mass-loss behavior, T5% and T10% were calculated from the original numerical TG data as the temperatures at which the residual mass decreased to 95% and 90%, respectively. The T5% values were 82.31, 106.62, 104.99, 82.44, and 92.23 °C for CS, LCS, 0.1% LLF-LCS, 0.2% LLF-LCS, and 0.3% LLF-LCS, respectively. The corresponding T10% values were 113.63, 136.05, 138.61, 118.21, and 129.06 °C. LCS and 0.1% LLF-LCS showed the highest T5% and T10% values, whereas the effects of the higher LLF concentrations were less pronounced, indicating a formulation-dependent response. Because the initial mass loss mainly involved the release of adsorbed water and other weakly retained volatile components, these parameters were interpreted as indicators of low-temperature mass-loss resistance rather than direct measures of polymer-matrix degradation stability [51]. The complete T5%, T10%, and residual-mass values at selected temperatures are provided in Table S3.

The principal degradation event occurred at approximately 250–350 °C, corresponding mainly to decomposition of the CS-rich composite matrix, followed by slower degradation and residue evolution at higher temperatures [51]. The DTG maximum increased from 282.2 °C for CS to 287.4 °C for LCS and to 289.9, 291.5, and 287.6 °C for the 0.1%, 0.2%, and 0.3% LLF-LCS films, respectively. Thus, the clearest improvement was a modest upward shift in the main degradation maximum, reaching a maximum increase of 9.3 °C for the 0.2% LLF-LCS film relative to CS, rather than a uniform enhancement of thermal stability over the entire 50–800 °C range. The LLF-containing films also retained 32.02–32.95% of their initial mass at 500 °C, compared with 28.02% for CS, indicating a higher high-temperature residue yield after LLF incorporation.

Similar improvements in thermal stability have been reported for gelatin–CS films containing *Cyclocarya paliurus* flavonoids and for CS films containing *Sauropus androgynus* extract [52,53]. In these systems, the altered thermal response was associated with interactions between the plant-derived components and the polymer matrix, including hydrogen bonding, which may contribute to matrix reorganization and delay the principal degradation event [52]. However, the effect of plant polyphenols on the thermal stability of CS films is formulation-dependent, because decreased thermal stability has also been reported after the incorporation of young apple and apple-peel polyphenols [33,54]. Taken together, the present results indicate that LY and LLF moderately increased resistance to the principal thermal-degradation event within approximately 250–350 °C, with the greatest effect observed for the 0.2% LLF-LCS formulation.

#### 3.3.5. Mechanical Strength and Extensibility of the Films

As illustrated in Figure 3C, the EB and TS values of LCS films were slightly lower than those of CS films; however, the differences were not statistically significant (*p* > 0.05). With the incorporation of LLF, both EB and TS of LLF-LCS films initially increased and then declined. At 0.2% LLF content, the films exhibited the highest EB, which was significantly greater than that of LCS films (*p* < 0.05). Similarly, TS also peaked at 0.2% LLF, showing a statistically significant improvement over the LCS group (*p* < 0.05). These enhancements in mechanical performance were likely attributed to the molecular interactions and cross-linking effects among LLF, LY, and CS within the film matrix.

### 3.4. Bioactivities of the Films

#### 3.4.1. Antibacterial Activity of the Films

Antibacterial activity is important for limiting microbial spoilage during food storage [54,55]. CS and LY act through different antibacterial mechanisms and have therefore been combined in antibacterial food-contact materials [6,7]. Spirescu et al. reported antiadhesive and antibiofilm activity for magnetite-based coatings containing CS- and LY-functionalized nanoparticles [56]. Although their system was developed for biomedical applications, it provides relevant support for the use of CS and LY in antibacterial coating materials.

As shown in Figure 3D, the LCS film produced a significantly larger inhibition zone against *S. aureus* than the CS film (*p* < 0.05), indicating that LY mainly enhanced antibacterial activity against the Gram-positive strain. In contrast, no significant improvement was observed against *E. coli* (*p* > 0.05), probably because the outer membrane of Gram-negative bacteria restricts LY access to the underlying peptidoglycan layer [6].

After LLF incorporation, the LLF-LCS films exhibited significantly larger inhibition zones against both *S. aureus* and *E. coli* than the LCS film (*p* < 0.05). This finding indicates that LLF broadened the antibacterial response of the coating, particularly against *E. coli*, which was less susceptible to LY alone. Flavonoid-rich extracts may inhibit bacteria through several mechanisms, including disruption of membrane integrity and permeability and interference with cellular enzymes and energy metabolism [57]. Similar increases in antibacterial activity have been reported after the incorporation of *Cyclocarya paliurus* flavonoids into gelatin–CS films [52].

The inhibition zones reflect both the antibacterial activity of the film components and their diffusion through the agar. Therefore, these results demonstrate the in vitro antibacterial potential of the LLF-LCS films, although their efficacy on coated strawberries remains to be verified.

#### 3.4.2. Antioxidant Activity of the Films

Antioxidant activity can help limit oxidative deterioration and is therefore an important property of active food-coating materials [58]. As shown in Figure 3E,F, no significant difference in DPPH or ABTS radical-scavenging activity was observed between the CS and LCS films. In contrast, both activities increased markedly after LLF incorporation, and all LLF-LCS films showed significantly higher values than the CS and LCS films (*p* < 0.05).

This enhancement was mainly attributed to the intrinsic radical-scavenging activity of LLF. Similar improvements in antioxidant activity have been reported after the incorporation of lotus leaf flavonoids or other plant flavonoids into CS-based composite films [17,52].

Overall, LLF incorporation improved both the in vitro antibacterial activity and antioxidant activity of the LCS system, supporting its potential as a bioactive coating for postharvest preservation.

### 3.5. Preservation Performance of LLF-LCS Coatings on Strawberries

#### 3.5.1. Visual Appearance and Physicochemical Quality During Storage

**Figure 4:**
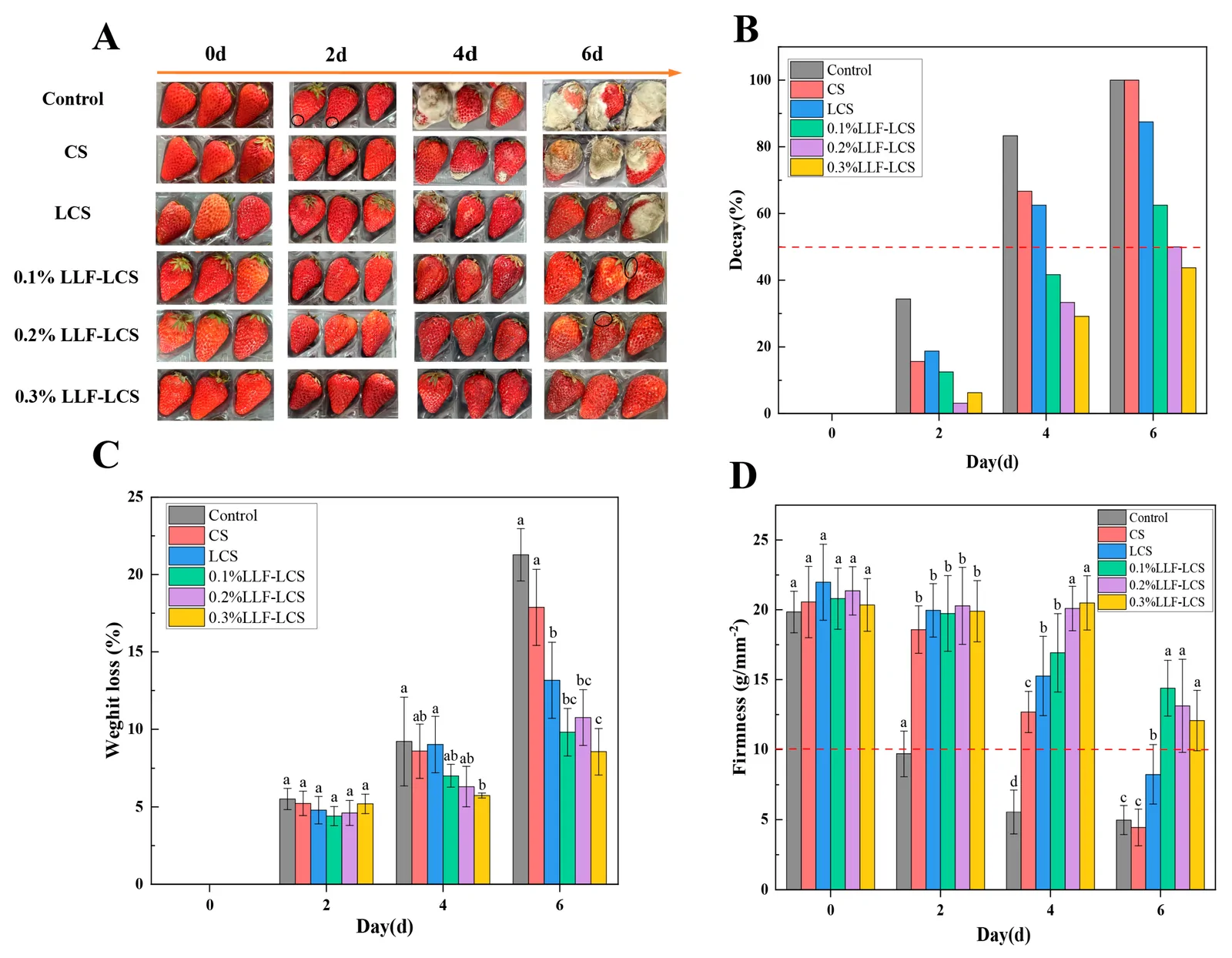
Preservation performance of LLF-LCS coatings on strawberries: (**A**) representative photographs; three fruits were randomly selected from the eight fruits in each treatment at each sampling time, and different fruits are shown because of destructive sampling; (**B**) decay incidence, calculated from eight fruits per treatment at each sampling time; (**C**) weight loss (*n* = 8 individual fruits); and (**D**) firmness (*n* = 5 individual fruits). Three puncture measurements were obtained from each fruit and averaged to provide one firmness value. Data are presented as mean ± SD. Different superscript letters indicate significant differences among treatments on the same storage day (*p* < 0.05). The red dashed lines in panels B and D indicate visual reference levels of 50% decay incidence and 10 g/mm^2^ firmness, respectively.

As shown in Figure 4A,B, decay incidence increased progressively in all groups during the 6-day storage period. Coated strawberries (CS and LCS) exhibited lower decay incidence than the uncoated control during the first 4 days, and LLF incorporation further reduced decay at later stages. By day 6, decay reached 100% in the control and CS groups, compared with 87.5% in LCS, 62.5% in 0.1% LLF-LCS, and <50% in both 0.2% and 0.3% LLF-LCS. These results indicate that LLF addition improved the anti-spoilage performance of the LCS coating under the tested conditions.

Weight loss increased over time in all treatments (Figure 4C), consistent with moisture loss and metabolic consumption during storage [59]. On day 6, the LCS and LLF-LCS groups showed significantly lower weight loss than the control and CS groups (*p* < 0.05), with the lowest value observed for 0.3% LLF-LCS (8.55%). Although film-level WVP increased slightly after LLF incorporation and was significantly higher at 0.3% LLF, this result is not necessarily inconsistent with the reduced weight loss of coated strawberries. WVP was measured using free-standing films under controlled conditions, whereas fruit weight loss reflects the behavior of a thin coating on a curved, biologically active surface and may also be affected by coating coverage, thickness, interactions with the native cuticle, and tissue deterioration. Therefore, the lower fruit weight loss should not be interpreted as evidence that LLF improved the intrinsic water-vapor barrier of the films.

Firmness decreased in all groups during storage (Figure 4D), reflecting softening associated with cell wall degradation, water loss, and spoilage progression [60]. The control group exhibited a rapid decline by day 2, and by day 6 the firmness of the control, CS, and LCS groups fell below 10 g/mm^2^. In contrast, LLF-LCS treatments retained significantly higher firmness (*p* < 0.05), remaining above 50% of the initial values. The improved firmness retention was consistent with the lower decay incidence and reduced dehydration observed in LLF-LCS-coated fruit.

#### 3.5.2. Antioxidant-Related Quality Attributes During Storage

As shown in Table 5, no significant differences in TSS were observed among treatments at day 2 (*p* > 0.05), whereas a more pronounced decline in TSS was observed on days 4 and 6. By day 6, strawberries in the 0.3% LLF-LCS group maintained TSS values comparable to those at day 0 (*p* > 0.05), indicating slower depletion of soluble solids under this treatment. TA remained generally stable in the coated groups throughout storage, while the control group exhibited a significant increase by day 6 (*p* < 0.05). This change may partially reflect an apparent concentration effect associated with greater dehydration and decay severity in the control fruit [61]. In addition, as indicated in Figure 4A,B, the control group experienced severe fungal contamination, which may have led to the production of acidic metabolites, thereby increasing overall acidity [62]. Consistently, pH trends broadly mirrored TA variations: a significant pH decrease was observed in the control group by the end of storage (*p* < 0.05), whereas coated groups showed only modest, non-significant changes (*p* > 0.05).

TPC, TAC and AAC showed a transient rise at day 2 across treatments (Table 5), which has been reported for strawberries stored at room temperature and is often attributed to early-stage physiological responses and concentration effects [63]. Thereafter, TPC decreased significantly in the control and CS groups by day 6 relative to day 0 (*p* < 0.05), which may be associated with oxidative/enzymatic degradation during storage [64]. In contrast, LCS-treated and LLF-LCS-treated fruit showed no significant loss of TPC. TAC declined sharply in the control from day 4 onward and dropped to less than half of the initial level by day 6, consistent with anthocyanin degradation during senescence and storage [65]. Significant reductions were also observed in the CS and LCS groups at day 6 (*p* < 0.05), while LLF-LCS treatments maintained comparatively stable TAC throughout storage. AAC decreased over time in all groups, in line with its susceptibility to oxidative loss under oxygen exposure and elevated storage temperature [66]. However, by day 6 the control and CS groups exhibited significantly lower AAC than the coated groups (*p* < 0.05). Overall, LLF-LCS coatings were associated with better preservation of phenolics, anthocyanins and ascorbic acid during storage, consistent with the improved quality retention observed in coated fruit (Section 3.5.1).

Together with the lower decay incidence and improved firmness retention, these results indicate that LLF-LCS coatings helped maintain both physical and chemical quality of strawberries during ambient storage.

Overall, 0.2% LLF-LCS was considered the most balanced formulation within the tested range rather than the best-performing formulation for every individual parameter. At this concentration, TS and EB reached their highest values, while WVP remained statistically comparable to those of the CS and LCS films. The 0.2% formulation also showed markedly improved antioxidant and in vitro antibacterial activities and preservation performance comparable to that of 0.3% LLF-LCS. Increasing the LLF concentration to 0.3% produced limited additional preservation benefits, while WVP and opacity increased and mechanical performance declined from the values observed at 0.2%. Thus, 0.2% LLF provided the most favorable compromise between material properties, bioactivity, and short-term strawberry preservation.

## 4. Conclusions

LLF incorporation effectively improved the functionality of LCS-based coatings. Structural analyses indicated that LLF was incorporated into the LCS matrix mainly through non-covalent interactions, resulting in formulation-dependent changes in matrix organization and physicochemical properties. Compared with the CS and LCS systems, the LLF-LCS formulations showed stronger antioxidant and in vitro antibacterial activities, supporting the contribution of LLF as a multifunctional active component.

In the strawberry trial, LLF-LCS coatings delayed deterioration during ambient storage by reducing decay and weight loss, maintaining firmness, and improving the retention of ascorbic acid, total phenolics, and anthocyanins. Considering both coating properties and preservation efficacy, the 0.2% LLF-LCS formulation provided the best overall balance, whereas the clearest preservation benefits were observed with the 0.2% and 0.3% treatments. These results support the use of purified lotus leaf flavonoids to improve conventional LCS coatings and demonstrate their potential in biodegradable postharvest preservation systems.

The preservation trial was limited to one strawberry cultivar from a single harvest lot under one ambient-storage condition. In addition, antibacterial efficacy on the fruit surface and sensory acceptance of the coatings were not evaluated directly; these aspects should be examined in future application-oriented studies.

## Figures and Tables

**Table 1:** Major flavonoid constituents of LLF characterized by UPLC-QTOF-MS/MS and their relative abundances.

Compound	Molecular Formula	Ion Mode	RT (min)	Precursor *m*/*z*	Mass Error (ppm)	Adduct	Total Score	Relative Peak Area (%)
Quercetin 3-O-glucuronide (miquelianin)	C_21_H_18_O_13_	ESI−	8.04085	477.0663	2.450387159	[M−H]−	91.8	17.89
Peltatoside	C_26_H_28_O_16_	ESI−	7.4752	595.1296	1.451782512	[M−H]−	96.8	12.35
Kaempferol 3-O-glucoside (astragalin)	C_21_H_20_O_11_	ESI+	9.379983	449.107	1.872601463	[M+H]+	94.1	6.29
Quercetin 3-O-arabinoside	C_20_H_18_O_11_	ESI−	8.762366	433.0777	0.140852343	[M−H]−	94.9	3.86
Quercetin 3-O-galactoside (hyperoside)	C_21_H_20_O_12_	ESI+	8.139584	465.1044	3.53470277	[M+H]+	92.2	3.78
Kaempferol	C_15_H_10_O_6_	ESI+	9.379983	287.0538	4.236121761	[M+H]+	96	3.32
Quercetin	C_15_H_10_O_7_	ESI+	8.139584	303.0515	5.177364650	[M+H]+	96.5	3.05
Isorhamnetin 3-O-galactoside	C_22_H_22_O_12_	ESI+	9.584617	479.1188	0.822343694	[M+H]+	92.3	2.78
Luteolin	C_15_H_10_O_6_	ESI−	11.78443	285.0388	5.837767651	[M−H]−	93.9	2.51
Myricetin 3-O-galactoside	C_21_H_20_O_13_	ESI−	7.3316	479.0844	2.673857519	[M−H]−	97.2	2.05
Rutin (quercetin 3-O-rutinoside)	C_27_H_30_O_16_	ESI−	7.841683	609.1469	1.290330812	[M−H]−	94.3	1.41
Isorhamnetin 3-O-rutinoside	C_28_H_32_O_16_	ESI−	9.0799	623.1627	1.502017701	[M−H]−	97.2	0.90

Note: Compounds were annotated based on accurate MS1 mass and MS/MS spectral matching against integrated databases. Relative peak areas were normalized separately within the positive- and negative-ion datasets and were used for semi-quantitative estimation of relative abundance. Values obtained in different ionization modes should not be directly compared as absolute concentrations.

**Table 2:** Antibacterial activity of LLF against *S. aureus* and *E. coli* (*n* = 3).

Strain	Inhibition Zone Diameter (mm) at LLF Concentration (mg/mL)						MIC (mg/mL)	MBC (mg/mL)
	20	15	10	5	2.5	1.25		
*S. aureus*	14.15 ± 0.34 ^a^	13.72 ± 0.26 ^a^	11.09 ± 0.10 ^b^	9.68 ± 0.15 ^c^	8.59 ± 0.06 ^de^	8.43 ± 0.10 ^e^	0.625	5
*E. coli*	11.36 ± 0.71 ^b^	9.95 ± 0.24 ^b^	9.37 ± 0.08 ^c^	9.20 ± 0.07 ^cd^	8.52 ± 0.19 ^de^	8.36 ± 0.06 ^e^	1.25	20

Note: Values are mean ± SD (*n* = 3). Different superscript letters within the same row indicate significant differences among concentrations (*p* < 0.05).

**Table 3:** Thickness, color, opacity, and appearance of different films (*n* = 3).

Films	Thickness (mm)	Color						Opacity (A·mm^−1^)	Appearance
		L*	a*	b*	ΔE	WI	YI		
CS	0.108 ± 0.007 ^b^	89.75 ± 0.08 ^a^	1.22 ± 0.04 ^d^	7.70 ± 0.38 ^d^	9.96 ± 0.34 ^d^	87.12 ± 0.29 ^a^	12.25 ± 0.62 ^a^	0.81 ± 0.09 ^a^	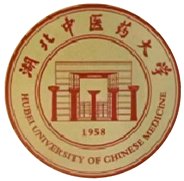
LCS	0.128 ± 0.022 ^ab^	83.86 ± 0.23 ^b^	1.59 ± 0.13 ^c^	30.38 ± 0.21 ^c^	32.58 ± 0.11 ^c^	65.56 ± 0.09 ^b^	51.75 ± 0.22 ^b^	1.14 ± 0.01 ^a^	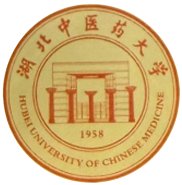
0.1% LLF-LCS	0.143 ± 0.016 ^a^	52.92 ± 0.77 ^d^	14.70 ± 0.70 ^b^	40.09 ± 0.95 ^b^	60.64 ± 1.02 ^ab^	36.44 ± 1.01 ^c^	108.23 ± 3.11 ^c^	1.38 ± 0.09 ^b^	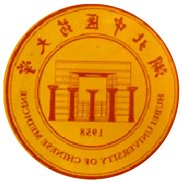
0.2% LLF-LCS	0.147 ± 0.019 ^a^	60.73 ± 0.30 ^c^	20.50 ± 0.30 ^a^	50.22 ± 0.01 ^a^	64.60 ± 0.27 ^a^	33.03 ± 0.28 ^c^	118.15 ± 0.63 ^c^	1.73 ± 0.03 ^c^	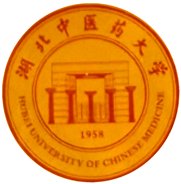
0.3% LLF-LCS	0.151 ± 0.015 ^a^	49.85 ± 1.18 ^d^	21.50 ± 0.29 ^a^	37.57 ± 1.90 ^bc^	63.29 ± 0.25 ^b^	33.73 ± 0.18 ^c^	107.62 ± 2.88 ^c^	1.86 ± 0.03 ^d^	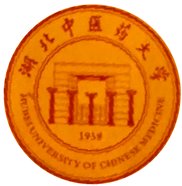

Note: Different letters in each column indicate significant differences between the films (*p* < 0.05).

**Table 4:** Water vapor permeability, oxygen permeability, carbon dioxide permeability, moisture content, water solubility, and water contact angle of films (*n* = 3).

	WVP × 10^13^ (kg·m·m^−2^·s^−1^·Pa^−1^)	OP × 10^14^ (kg·m·m^−2^·s^−1^·Pa^−1^)	CDP × 10^11^ (kg·m·m^−2^·s^−1^·Pa^−1^)	CDP/OP × 10^3^	MC (%)	WS (%)	WCA (°)	Image
CS	2.78 ± 0.18 ^b^	1.32 ± 0.15 ^a^	1.64 ± 0.05 ^b^	1.24 ± 0.10 ^b^	29.63 ± 0.53 ^a^	17.75 ± 1.03 ^ab^	90.67 ± 0.50 ^a^	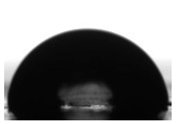
LCS	2.69 ± 0.12 ^b^	1.36 ± 0.13 ^a^	1.89 ± 0.10 ^ab^	1.38 ± 0.08 ^a^	27.34 ± 0.25 ^b^	15.75 ± 0.41 ^b^	73.27 ± 0.42 ^b^	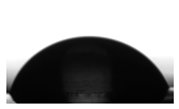
0.1% LLF-LCS	2.98 ± 0.06 ^ab^	1.41 ± 0.07 ^a^	1.97 ± 0.11 ^a^	1.39 ± 0.05 ^a^	23.15 ± 0.29 ^c^	18.20 ± 0.35 ^a^	80.80 ± 0.78 ^c^	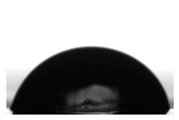
0.2% LLF-LCS	3.00 ± 0.06 ^ab^	1.54 ± 0.03 ^a^	2.16 ± 0.07 ^a^	1.40 ± 0.09 ^a^	23.34 ± 0.11 ^c^	18.79 ± 1.32 ^a^	88.43 ± 0.31 ^d^	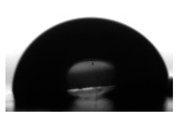
0.3% LLF-LCS	3.20 ± 0.12 ^a^	1.55 ± 0.07 ^a^	2.20 ± 0.13 ^a^	1.42 ± 0.06 ^a^	19.95 ± 0.34 ^d^	19.26 ± 0.38 ^a^	84.03 ± 0.25 ^e^	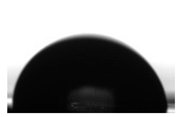

Note: Values are presented as mean ± SD. Different letters in each column indicate significant differences between the films (*p* < 0.05).

**Table 5:** TSS, TA, pH, TPC, TAC, and AAC of strawberries during 6 d of ambient storage.

	TSS (°Brix)	TA (mg Citric Acid 100 g^−1^)	pH	TPC (mg GAE 100 g^−1^)	TAC (mg Cyanidin-3-Glucoside 100 g^−1^)	AAC (mg Citric Acid 100 g^−1^)
Control						
0	1.93 ± 0.06 ^Aa^	0.58 ± 0.008 ^Bc^	3.86 ± 0.02 ^Bc^	124.18 ± 0.16 ^Aa^	44.69 ± 0.65 ^Aa^	37.62 ± 0.18 ^Bb^
2	1.77 ± 0.06 ^Bb^	0.70 ± 0.019 ^Ab^	3.69 ± 0.01 ^Da^	119.38 ± 0.33 ^Bb^	43.42 ± 1.34 ^Aa^	49.46 ± 0.25 ^Ba^
4	1.37 ± 0.06 ^Bc^	0.72 ± 0.021 ^Aab^	3.78 ± 0.01 ^CDb^	94.86 ± 0.17 ^Fc^	28.91 ± 1.44 ^Cb^	25.31 ± 0.64 ^Cd^
6	1.33 ± 0.06 ^Cc^	0.77 ± 0.032 ^Aa^	3.79 ± 0.02 ^BCb^	86.82 ± 0.42 ^Cd^	27.20 ± 0.42 ^Db^	26.82 ± 0.38 ^Cc^
CS						
0	1.97 ± 0.06 ^Aa^	0.60 ± 0.017 ^Bb^	3.81 ± 0.01 ^Ca^	104.07 ± 0.16 ^Da^	39.48 ± 0.42 ^Bb^	40.30 ± 0.41 ^Ab^
2	1.90 ± 0.10 ^Aa^	0.73 ± 0.002 ^Aa^	3.79 ± 0.02 ^Ba^	110.41 ± 0.16 ^Cb^	42.97 ± 0.19 ^Aa^	49.59 ± 0.25 ^Ba^
4	1.67 ± 0.06 ^Ab^	0.69 ± 0.031 ^Aab^	3.80 ± 0.04 ^Ca^	100.91 ± 0.17 ^Ec^	36.21 ± 0.50 ^Bc^	49.42 ± 0.25 ^Ba^
6	1.50 ± 0.10 ^BCb^	0.63 ± 0.010 ^Bb^	3.82 ± 0.02 ^Ca^	97.16 ± 0.16 ^Bd^	33.28 ± 0.70 ^Cd^	14.79 ± 0.06 ^Dc^
LCS						
0	2.13 ± 0.15 ^Aa^	0.66 ± 0.008 ^Bb^	3.93 ± 0.01 ^Aa^	107.77 ± 1.61 ^CDb^	40.60 ± 0.53 ^Ba^	39.60 ± 0.40 ^Aa^
2	1.86 ± 0.06 ^Bb^	0.55 ± 0.004 ^Cd^	3.85 ± 0.01 ^Bb^	123.08 ± 0.16 ^Aa^	45.16 ± 1.48 ^Aa^	51.73 ± 0.29 ^Aa^
4	1.67 ± 0.06 ^Abc^	0.70 ± 0.008 ^Aa^	3.72 ± 0.02 ^Db^	108.80 ± 0.33 ^Db^	38.15 ± 0.63 ^Bb^	51.55 ± 0.29 ^Ab^
6	1.57 ± 0.06 ^Bc^	0.58 ± 0.008 ^Cc^	3.78 ± 0.03 ^BCb^	105.10 ± 1.37 ^Bb^	36.40 ± 0.33 ^Bb^	34.91 ± 0.29 ^Bc^
0.1% LLF-LCS						
0	1.90 ± 0.10 ^Aa^	0.70 ± 0.002 ^Aa^	3.76 ± 0.01 ^Db^	99.99 ± 0.42 ^Eb^	33.36 ± 1.77 ^Ca^	38.45 ± 0.04 ^Aa^
2	2.03 ± 0.06 ^Aa^	0.59 ± 0.063 ^Ab^	3.81 ± 0.01 ^Ca^	115.80 ± 0.59 ^BCa^	38.18 ± 1.39 ^Ba^	52.06 ± 0.40 ^Aa^
4	1.67 ± 0.06 ^Ab^	0.68 ± 0.013 ^Ba^	3.74 ± 0.01 ^CDb^	110.53 ± 0.33 ^Cb^	36.96 ± 0.11 ^Ba^	51.89 ± 0.40 ^Ab^
6	1.60 ± 0.10 ^Bb^	0.65 ± 0.004 ^Ba^	3.85 ± 0.02 ^BCa^	105.56 ± 1.27 ^Bb^	35.25 ± 0.50 ^Ba^	34.18 ± 0.14 ^Bc^
0.2%LLF-LCS						
0	1.87 ± 0.06 ^Aa^	0.70 ± 0.005 ^Aa^	3.79 ± 0.01 ^Cba^	108.61 ± 0.32 ^Cd^	39.37 ± 1.09 ^Bb^	39.18 ± 0.26 ^Aa^
2	1.83 ± 0.06 ^Ba^	0.54 ± 0.013 ^BCb^	3.92 ± 0.01 ^Aa^	117.12 ± 0.59 ^Bc^	44.86 ± 1.39 ^Aa^	52.52 ± 0.97 ^Aa^
4	1.83 ± 0.06 ^Aa^	0.51 ± 0.008 ^Db^	4.03 ± 0.03 ^Aa^	129.85 ± 0.17 ^Ab^	45.05 ± 0.67 ^Aa^	52.34 ± 0.96 ^Ab^
6	1.67 ± 0.06 ^Ab^	0.54 ± 0.002 ^Cb^	4.02 ± 0.01 ^Aa^	126.71 ± 0.64 ^Aa^	44.75 ± 0.95 ^Aa^	39.68 ± 0.18 ^Ab^
0.3%LLF-LCS						
0	1.93 ± 0.06 ^Aa^	0.62 ± 0.005 ^Cb^	3.86 ± 0.01 ^Ba^	112.69 ± 0.48 ^Ba^	41.04 ± 0.51 ^Bb^	38.32 ± 0.16 ^Aba^
2	1.97 ± 0.06 ^Aa^	0.58 ± 0.005 ^Bb^	3.85 ± 0.01 ^Bab^	114.10 ± 0.16 ^BCa^	43.94 ± 0.57 ^Aa^	48.13 ± 0.61 ^Ba^
4	1.80 ± 0.10 ^Aa^	0.58 ± 0.005 ^Cb^	3.86 ± 0.02 ^Bab^	126.00 ± 0.17 ^Bb^	44.57 ± 0.36 ^Aa^	47.97 ± 0.61 ^Bb^
6	1.86 ± 0.06 ^Aa^	0.65 ± 0.005 ^Bb^	3.89 ± 0.01 ^Ba^	123.76 ± 1.20 ^Ab^	43.94 ± 0.74 ^Aa^	40.89 ± 0.79 ^Ac^

Note: Values are presented as mean ± SD of three independently prepared subsamples obtained from pooled tissue of eight fruits per treatment at each sampling time. Different uppercase letters indicate significant differences among treatments at the same storage time, whereas different lowercase letters indicate significant differences among storage times within the same treatment (*p* < 0.05).

## Data Availability

The original contributions presented in this study are included in the article/Supplementary Materials. Further inquiries can be directed to the corresponding author.

## Supplementary Materials

The following supporting information can be downloaded at: https://www.mdpi.com/article/10.3390/foods15173073/s1, Figure S1: Total ion chromatograms (TICs) of purified lotus leaf flavonoids (LLF) obtained by UPLC-QTOF-MS/MS in (A) positive electrospray ionization (ESI+) mode and (B) negative electrospray ionization (ESI−) mode; Figure S2: Three-component fitting of the C–H stretching region at 3000–2800 cm^−1^ in CS, LCS, and LLF-LCS films; Table S1: Detailed mass-spectrometric information of LLF; Table S2: Semi-quantitative ATR-FTIR parameters of the O–H/N–H and C–H stretching regions in CS, LCS, and LLF-LCS films; Table S3: Initial mass-loss temperatures and residual masses of CS, LCS, and LLF-LCS films.
