# Supplementary material for: Purified Lotus Leaf Flavonoids in Lysozyme–Chitosan Coatings: Effects on Coating Properties and Strawberry Preservation
Source: Foods. 2026 Aug 30;15(17):3073. doi: 10.3390/foods15173073 (PMC13565157; doi:10.3390/foods15173073)
Supplement: Supplementary file 1 [file foods-15-03073-s001.zip › foods-4435752-supplementary.pdf]

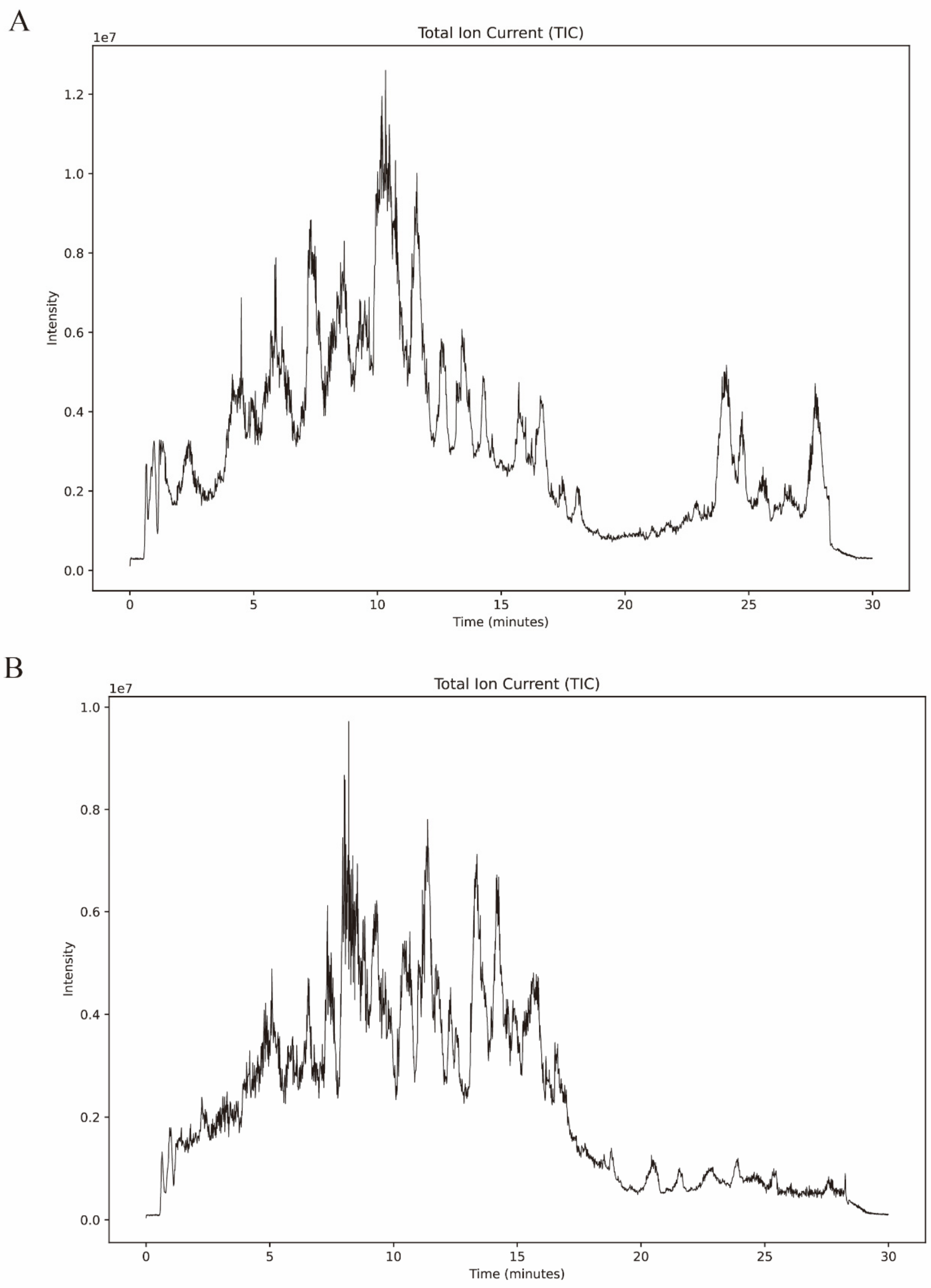

Figure S1. Total ion chromatograms (TICs) of purified lotus leaf flavonoids (LLF) obtained by UPLC-QTOF-MS/MS in (A) positive electrospray ionization (ESI+) mode and (B) negative electrospray ionization (ESI−) mode. Detailed annotations of the detected flavonoids are provided in Table S1.

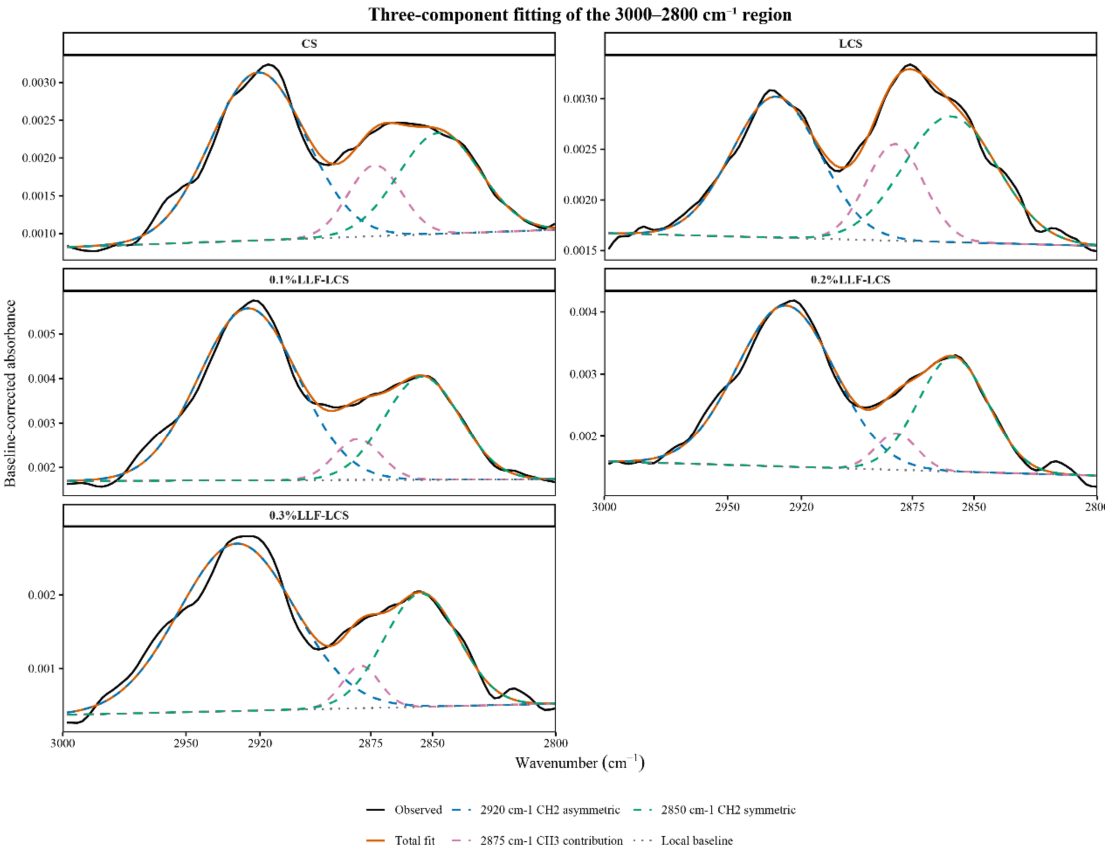

Figure S2. Three-component fitting of the C–H stretching region at 3000–2800 cm^{-1} in CS, LCS, and LLF-LCS films. The baseline-corrected experimental spectra are shown as black solid lines, and the total fitted spectra are shown as orange solid lines. The dashed components represent the bands assigned approximately to CH_2 asymmetric stretching near 2920 cm^{-1}, a possible CH_3 stretching contribution near 2875 cm^{-1}, and CH_2 symmetric stretching near 2850 cm^{-1}. Grey dotted lines indicate the fitted local baselines. The fitted components were used to calculate the descriptive $A_{2875}/(A_{2920} + A_{2850})$ area index reported in Table S2. Owing to spectral overlap and the model dependence of peak fitting, this index should not be interpreted as an absolute molecular CH_3/CH_2 ratio.

Table S1 Detailed mass-spectrometric information of LLF.

No.	Standardized compound name	Original software annotation	Molecular formula	Ion mode	RT (min)	Observed precursor m/z	Theoretical m/z	Mass error (ppm)	Adduct	Dot product	Reverse dot product	Fragment presence (%)	Total score	Relative peak area (%)
1	Quercetin 3-O-glucuronide (miquelianin)	Miquelianin	$C_{21}H_{18}O_{13}$	ESI+	8.155	479.0841	479.0820	4.34	[M+H]+	86.3	86.2	100.0	91.7	14.65
2	Kaempferol 3-O-glucoside (astragalin)	Astragalin	$C_{21}H_{20}O_{11}$	ESI+	9.380	449.1070	449.1078	1.87	[M+H]+	91.4	87.0	100.0	94.1	6.29
3	Quercetin 3-O-apiosyl-(1→2)-galactoside	Quercetin 3-O-apiosyl-(1->2)-galactoside	$C_{26}H_{28}O_{16}$	ESI+	7.582	597.1457	597.1450	1.15	[M+H]+	99.5	76.5	100.0	94.6	5.72
4	Quercetin 3-O-galactoside (hyperoside)	Hyperin	$C_{21}H_{20}O_{12}$	ESI+	8.140	465.1044	465.1028	3.53	[M+H]+	90.5	82.9	96.3	92.2	3.78
5	Kaempferol	Kaempferol	$C_{15}H_{10}O_{6}$	ESI+	9.380	287.0538	287.0550	4.24	[M+H]+	89.8	98.3	100.0	96.0	3.32
6	Procyanidin B2	Procyanidin B2	$C_{30}H_{26}O_{12}$	ESI+	4.889	579.1486	579.1497	1.91	[M+H]+	83.0	95.3	97.6	92.9	3.30
7	Rhamnetin	Rhamnetin	$C_{16}H_{12}O_{7}$	ESI+	11.504	317.0665	317.0656	2.90	[M+H]+	80.0	84.7	81.5	87.7	3.23
8	Quercetin	Quercetin	$C_{15}H_{10}O_{7}$	ESI+	8.140	303.0515	303.0499	5.18	[M+H]+	94.1	97.5	95.2	96.5	3.05
9	Isorhamnetin 3-O-galactoside	Isorhamnetin 3-galactoside	$C_{22}H_{22}O_{12}$	ESI+	9.585	479.1188	479.1184	0.82	[M+H]+	89.8	87.5	86.2	92.3	2.78
10	Jaceoside	Jaceoside	$C_{23}H_{24}O_{12}$	ESI+	12.600	493.1337	493.1341	0.72	[M+H]+	81.6	87.9	100.0	91.2	2.36
11	Epicatechin	Epicatechin	$C_{15}H_{14}O_{6}$	ESI+	5.320	291.0868	291.0863	1.66	[M+H]+	95.0	97.8	100.0	97.8	2.15
12	Isorhamnetin	Isorhamnetin	$C_{16}H_{12}O_{7}$	ESI+	9.600	317.0661	317.0656	1.64	[M+H]+	86.0	95.3	93.3	93.5	2.06

13	Nobiletin	Nobiletin	$C_{21}H_{22}O_8$	ESI+	16.616	403.1371	403.1387	4.08	[M+H]+	90.9	91.8	88.4	93.4	1.71
14	Luteolin	Luteolin	$C_{15}H_{10}O_6$	ESI+	11.901	287.0537	287.0550	4.58	[M+H]+	93.1	92.2	97.4	95.4	1.37
15	Rhamnazin	Rhamnazin	$C_{17}H_{14}O_7$	ESI+	12.600	331.0797	331.0812	4.62	[M+H]+	78.2	85.2	63.2	85.0	1.33
16	Tangeritin	Tangeritin	$C_{20}H_{20}O_7$	ESI+	17.509	373.1285	373.1282	0.85	[M+H]+	76.8	93.3	100.0	90.4	1.29
17	Guaiaverin	Guaiaverin	$C_{20}H_{18}O_{11}$	ESI+	8.967	435.0925	435.0922	0.71	[M+H]+	88.0	83.3	84.6	90.6	1.03
18	Isorhamnetin 3-O-neohesperidoside	Isorhamnetin 3-neohesperidoside	$C_{28}H_{32}O_{16}$	ESI+	10.790	625.1783	625.1763	3.17	[M+H]+	88.1	88.0	100.0	92.9	1.00
19	4',5,7,8-Tetramethoxyflavone	4',5,7,8-Tetramethoxyflavone	$C_{19}H_{18}O_6$	ESI+	16.727	343.1177	343.1176	0.24	[M+H]+	81.4	93.0	95.2	91.7	0.83
20	5,6,7,3',4'-Pentamethoxyflavone	5,6,7,3',4'-Pentamethoxyflavone	$C_{20}H_{20}O_7$	ESI+	15.712	373.1275	373.1282	1.83	[M+H]+	82.2	92.0	95.0	91.7	0.83
21	Rutin (quercetin 3-O-rutinoside)	Rutin	$C_{27}H_{30}O_{16}$	ESI+	7.980	611.1639	611.1607	5.29	[M+H]+	85.9	91.8	100.0	92.4	0.74
22	Andrographidin B	Andrographidin B	$C_{23}H_{24}O_{12}$	ESI+	10.774	493.1333	493.1341	1.53	[M+H]+	81.0	79.5	92.9	88.2	0.47
23	Sternbin	Sternbin	$C_{16}H_{14}O_6$	ESI+	12.358	303.0861	303.0863	0.71	[M+H]+	70.6	77.8	63.2	81.2	0.44
24	Artemisetin	Artemisetin	$C_{20}H_{20}O_8$	ESI+	18.212	389.1215	389.1231	4.10	[M+H]+	70.7	79.8	64.7	81.4	0.42
25	Syringetin 3-O-galactoside	Syringetin 3-galactoside	$C_{23}H_{24}O_{13}$	ESI+	9.585	509.1262	509.1290	5.44	[M+H]+	87.4	82.9	94.1	90.1	0.41
26	5-Hydroxy-2',4',7,8-Tetramethoxyflavone	5-Hydroxy-2',4',7,8-Tetramethoxyflavone	$C_{19}H_{18}O_7$	ESI+	17.456	359.1139	359.1125	3.81	[M+H]+	70.1	89.7	100.0	86.7	0.36

27	Kaempferol 7-O-neohesperidoside	Kaempferol-7-O-neohesperidoside	$C_{27}H_{30}O_{15}$	ESI+	9.030	595.1644	595.1658	2.27	[M+H]+	89.4	90.4	100.0	94.1	0.31
28	Myricetin 3-O-galactoside	Myricetin 3-galactopyranoside	$C_{21}H_{20}O_{13}$	ESI+	7.444	481.0970	481.0977	1.39	[M+H]+	87.8	86.9	63.6	88.9	0.26
29	Epigallocatechin	Epigallocatechin	$C_{15}H_{14}O_{7}$	ESI+	3.679	307.0827	307.0812	4.78	[M+H]+	77.1	80.9	69.2	84.3	0.25
30	Diosmetin	Diosmetin	$C_{16}H_{12}O_{6}$	ESI+	12.324	301.0701	301.0707	1.88	[M+H]+	65.4	79.5	100.0	83.9	0.19
31	3,3',4',5,6,7,8-Heptamethoxyflavone	3,3',4',5,6,7,8-Heptamethoxyflavone	$C_{22}H_{24}O_{9}$	ESI+	17.131	433.1486	433.1493	1.64	[M+H]+	71.4	90.5	100.0	88.3	0.18
32	Leucoside	Leucoside	$C_{26}H_{28}O_{15}$	ESI+	8.292	581.1548	581.1501	8.09	[M+H]+	81.9	78.3	87.5	85.1	0.14
33	Gardenin	Gardenin	$C_{21}H_{22}O_{9}$	ESI+	17.502	419.1331	419.1337	1.34	[M+H]+	70.3	88.0	69.2	83.9	0.14
34	Neoliquiritin	Neoliquiritin	$C_{21}H_{22}O_{9}$	ESI+	10.728	419.1328	419.1337	2.05	[M+H]+	76.4	95.9	100.0	91.1	0.09
35	Chrysoeriol	Chrysoeriol	$C_{16}H_{12}O_{6}$	ESI+	9.901	301.0700	301.0707	2.21	[M+H]+	83.5	93.1	100.0	92.9	0.08
36	Salvigenin	Salvigenin	$C_{18}H_{16}O_{6}$	ESI+	18.354	329.1010	329.1020	2.94	[M+H]+	90.7	82.6	52.9	87.7	0.08
37	Kaempferol 3-O-α-L-arabinoside	Kaempferol 3-.alpha.-L-arabinoside	$C_{20}H_{18}O_{10}$	ESI+	9.776	419.0957	419.0973	3.76	[M+H]+	74.1	85.2	80.0	85.4	0.08
38	Cosmosiin	Cosmosiin	$C_{21}H_{20}O_{10}$	ESI+	9.635	433.1083	433.1129	10.68	[M+H]+	78.5	86.7	100.0	86.5	0.07
39	Demethoxycentaureidin 7-O-rutinoside	Demethoxycentaureidin 7-O-rutinoside	$C_{29}H_{34}O_{16}$	ESI+	11.692	639.1959	639.1920	6.15	[M+H]+	69.4	83.2	100.0	84.6	0.07

40	Neodiosmin	Neodiosmin	$C_{28}H_{32}O_{15}$	ESI+	9.729	609.1840	609.1814	4.27	[M+H]+	80.9	76.9	66.7	84.3	0.06
41	Cirsimarin	Cirsimarin	$C_{23}H_{24}O_{11}$	ESI+	10.759	477.1405	477.1391	2.85	[M+H]+	75.8	76.7	50.0	80.9	0.05
42	Quercetin 3-O-glucuronide (miquelianin)	Miquelianin	$C_{21}H_{18}O_{13}$	ESI−	8.041	477.0663	477.0675	2.45	[M−H] −	81.0	92.6	100.0	91.8	17.89
43	Peltatoside	Peltatoside	$C_{26}H_{28}O_{16}$	ESI−	7.475	595.1296	595.1305	1.45	[M−H] −	95.6	95.0	95.2	96.8	12.35
44	Kaempferol 3-O-glucoside (astragalin)	Astragalin	$C_{21}H_{20}O_{11}$	ESI−	9.236	447.0910	447.0933	5.12	[M−H] −	73.6	77.5	100.0	85.3	9.97
45	Quercetin	Quercetin	$C_{15}H_{10}O_{7}$	ESI−	11.784	301.0340	301.0354	4.58	[M−H] −	90.0	95.8	90.9	94.4	5.76
46	Scutellarin	Scutellarin	$C_{21}H_{18}O_{12}$	ESI−	9.282	461.0704	461.0726	4.67	[M−H] −	68.9	91.3	100.0	87.0	4.97
47	Procyanidin C1	Procyanidin C1	$C_{45}H_{38}O_{18}$	ESI−	6.638	865.2025	865.1985	4.57	[M−H] −	86.0	68.1	60.4	83.0	4.57
48	Quercetin 3-O-arabinoside	Quercetin-3-Arabinoside	$C_{20}H_{18}O_{11}$	ESI−	8.762	433.0777	433.0776	0.14	[M−H] −	88.1	94.9	100.0	94.9	3.86
49	Luteolin	Luteolin	$C_{15}H_{10}O_{6}$	ESI−	11.784	285.0388	285.0405	5.84	[M−H] −	89.9	95.4	88.9	93.9	2.51
50	Isorhamnetin 3-O-galactoside-6″-O-rhamnoside	isorhamnetin-3-O-galactoside-6"-rhamnoside	$C_{28}H_{32}O_{16}$	ESI−	10.656	623.1611	623.1618	1.07	[M−H] −	94.8	93.7	83.3	95.0	2.28
51	Myricetin 3-O-galactoside	Myricetin-3-Galactoside	$C_{21}H_{20}O_{13}$	ESI−	7.332	479.0844	479.0831	2.67	[M−H] −	93.8	98.1	100.0	97.2	2.05
52	Isorhamnetin 3-O-galactoside	Isorhamnetin 3-galactoside	$C_{22}H_{22}O_{12}$	ESI−	9.453	477.1129	477.1039	18.96	[M−H] −	86.1	90.8	93.3	81.5	1.88

53	Quercetin 3-O-galactoside (hyperoside)	Hyperoside	$C_{21}H_{20}O_{12}$	ESI−	7.980	463.0842	463.0882	8.65	[M−H] −	90.5	97.6	100.0	94.4	1.44
54	Rutin (quercetin 3-O-rutinoside)	Quercetin-3-O-rutinoside	$C_{27}H_{30}O_{16}$	ESI−	7.842	609.1469	609.1461	1.29	[M−H] −	92.4	91.5	88.9	94.3	1.41
55	Isorhamnetin 3-O-rutinoside	Isorhamnetin-3-O-rutinoside	$C_{28}H_{32}O_{16}$	ESI−	9.080	623.1627	623.1618	1.50	[M−H] −	98.6	94.8	90.0	97.2	0.90
56	Gallocatechin	Gallocatechin	$C_{15}H_{14}O_{7}$	ESI−	3.623	305.0666	305.0667	0.26	[M−H] −	87.4	81.6	62.5	87.6	0.71
57	Jaceoside	Jaceoside	$C_{23}H_{24}O_{12}$	ESI−	10.671	491.1211	491.1195	3.25	[M−H] −	82.5	72.8	72.7	84.7	0.66
58	Hovetrichoside C	Hovetrichoside C	$C_{21}H_{22}O_{11}$	ESI−	6.370	449.1062	449.1089	6.10	[M−H] −	80.5	91.6	41.2	84.0	0.64
59	Kaempferol 3-O-rutinoside	Kaempferol-3-O-rutinoside	$C_{27}H_{30}O_{15}$	ESI−	8.919	593.1489	593.1512	3.88	[M−H] −	94.9	96.1	72.7	93.8	0.63
60	Quercitrin	Quercitrin	$C_{21}H_{20}O_{11}$	ESI−	9.984	447.0932	447.0933	0.20	[M−H] −	50.1	95.2	100.0	82.3	0.56
61	Syringetin 3-O-galactoside	Syringetin-3-O-galactoside	$C_{23}H_{24}O_{13}$	ESI−	9.469	507.1143	507.1144	0.23	[M−H] −	85.9	95.8	93.8	93.7	0.54
62	Procyanidin B2	Procyanidin B2	$C_{30}H_{26}O_{12}$	ESI−	1.016	577.1360	577.1352	1.47	[M−H] −	77.0	91.9	100.0	90.4	0.46
63	(-)-Catechin	(-)-Catechin	$C_{15}H_{14}O_{6}$	ESI−	6.273	289.0713	289.0718	1.61	[M−H] −	90.7	84.6	65.5	89.6	0.44
64	Sinensin	Sinensin	$C_{21}H_{22}O_{11}$	ESI−	6.007	449.1100	449.1089	2.36	[M−H] −	67.6	86.4	50.0	80.4	0.37
65	Phloridzin	Phloridzin	$C_{21}H_{24}O_{10}$	ESI−	9.952	435.1293	435.1297	0.86	[M−H] −	71.2	85.8	85.7	85.6	0.33
66	Chrysoeriol	Chrysoeriol	$C_{16}H_{12}O_{6}$	ESI−	13.566	299.0536	299.0561	8.41	[M−H] −	73.6	84.9	100.0	86.8	0.28
67	(-)-Aromadendrin	(-)-Aromadendrin	$C_{15}H_{12}O_{6}$	ESI−	9.744	287.0549	287.0561	4.23	[M−H] −	88.3	86.2	56.2	87.9	0.28

68	Spiraeoside	Spiraeoside	$C_{21}H_{20}O_{12}$	ESI−	10.049	463.0876	463.0882	1.30	[M−H] −	58.2	97.2	85.7	83.9	0.27
69	Prunin	Prunin	$C_{21}H_{22}O_{10}$	ESI−	9.422	433.1119	433.1140	4.90	[M−H] −	89.0	91.1	66.7	89.9	0.25
70	Flavanomarein	Flavanomarein	$C_{21}H_{22}O_{11}$	ESI−	9.649	449.1069	449.1089	4.54	[M−H] −	67.8	88.6	100.0	86.4	0.20
71	Kaempferol 3-O-α-L-arabinoside	Kaempferol-3-O-alpha-L-arabinoside	$C_{20}H_{18}O_{10}$	ESI−	9.697	417.0835	417.0827	1.86	[M−H] −	82.7	91.2	71.4	89.0	0.13
72	Myricetin 3-O-xyloside	Myricetin-3-Xyloside	$C_{20}H_{18}O_{12}$	ESI−	7.636	449.0714	449.0726	2.57	[M−H] −	81.8	98.6	100.0	93.4	0.11
73	Rutin (quercetin 3-O-rutinoside)	Rutin	$C_{27}H_{30}O_{16}$	ESI−	8.557	609.1442	609.1461	3.14	[M−H] −	49.2	93.9	100.0	81.3	0.10

Note: Compounds were annotated by matching accurate MS1 mass and MS/MS spectra against the integrated METLIN, MassBank, MoNA, and HMDB databases. RT, retention time; ESI+, positive electrospray ionization mode; ESI−, negative electrospray ionization mode. Relative peak areas were normalized separately within each ionization mode and are reported as semi-quantitative estimates of relative abundance; values obtained in ESI+ and ESI− modes should not be directly compared as absolute concentrations.

Table S2. Semi-quantitative ATR-FTIR parameters of the O–H/N–H and C–H stretching regions in CS, LCS, and LLF-LCS films.

**Group**	**O–H/N–H band maximum (cm^{-1})**	**FWHM (cm^{-1})**	**O–H/N–H-to-fingerprint area index**	**CH_3/CH_2 area index**
CS	3361	310	0.128	0.156
LCS	3278	318	0.095	0.230
0.1% LLF-LCS	3304	251	0.123	0.076
0.2% LLF-LCS	3365	286	0.116	0.063
0.3% LLF-LCS	3198	288	0.143	0.057

Note: The O–H/N–H-to-fingerprint area index was calculated as the integrated area at 3500–3100 cm^{-1} divided by that at 1800–900 cm^{-1}. The CH_3/CH_2 area index was calculated as $A_{2875}/(A_{2920} + A_{2850})$ based on three-component Gaussian fitting of the 3000–2800 cm^{-1} region. These parameters are descriptive, semi-quantitative spectral indicators and do not represent absolute hydrogen-bond content or molecular CH_3/CH_2 ratios. No inferential statistical analysis was applied to the derived spectral parameters.

Table S3. Initial mass-loss temperatures and residual masses of CS, LCS, and LLF-LCS films.

Group	T5% (°C)	T10% (°C)	Residual mass at 110 °C (%)	Residual mass at 250 °C (%)	Residual mass at 500 °C (%)	Residual mass at 800 °C (%)
CS	82.31	113.63	90.54	62.76	28.06	24.99
LCS	106.62	136.05	94.56	62.01	29.29	25.38
0.1% LLF-LCS	104.99	138.61	94.38	66.96	32.96	28.90
0.2% LLF-LCS	82.44	118.21	91.04	63.60	32.02	27.73
0.3% LLF-LCS	92.23	129.06	92.56	65.43	32.34	28.16

Note: T5% and T10% represent the temperatures at which the residual mass first decreased to 95% and 90% of the initial mass, respectively. All values were calculated from the original numerical TG data by linear interpolation between the two adjacent measurement points surrounding each threshold or selected temperature. The values are descriptive results obtained from the representative TG curves, and no inferential statistical analysis was applied.
